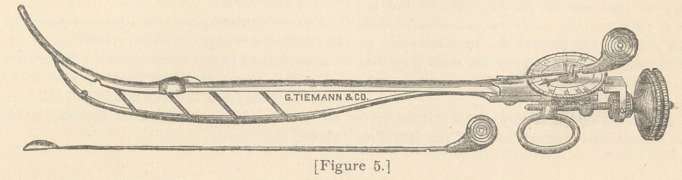# The American Medical Association

**Published:** 1887-07

**Authors:** 


### THE AMERICAN MEDICAL ASSOCIATION.

### THIRTY-EIGHTH ANNUAL MEETING.

The thirty eighth annual meeting of the American Medical Association was called to order in Central Music Hall, Chicago, at 11:05 A. M., Tuesday, June 7th, by Dr. Charles Gilman Smith, Chairman of the Committee of Arrangements. The following members were in attendance:

ALABAMA.

Wm. C. Cross, Tuscaloosa, Ala.
John M. Armstrong, Birmingham, Ala.
H. W. Blair, Sheffield, Ala.
Offa L. Shivers, Marvin, Ala.

ARKANSAS.

Wm. B. Lawrence, Batesville, Ark.
R. G. Jennings, Little Rock, Ark.
Ed. Cross, Little Rock, Ark.
W. H. Hawkins, Texarkana, Ark.
Thomas Edgar Murrell, Little Rock, Ark.
P. Van Patten, Forest City, Ark.
D. C. Ewing, Batesville, Ark.

CALIFORNIA.

Albert L. Gihon, Vallejo, Cal.
John W. Robertson, Crescent City, Cal.

COLORADO.

Peter Brumund, Idaho Springs, Colo.
Mark H. Sears, Leadville, Col.
Arnold Stedman, Denver, Col.

CONNECTICUT.

Edwin A. Hill, East Killingly, Conn.
Walter T. Barber, Waterbury, Conn.
Alfred North, Waterbury, Conn.
Lewis Barnes, Oxford, Conn.
Frank H. Whittemore, New Haven, Conn.

DAKOTA.

E. M. Darrow, Fargo, Dak.
Wm. E. Duncan, Ellendale, Dakota.
J. B. Van Velsor, Yankton, Dakota.
Isaac N. Wear, Fargo, Dak.
Berthold M. J. Conlin, Alexandria, Dak.

DISTRICT OF COLUMBIA.

Theoran Woolverton, Washington, D. C.
Robert Reyburn, Washington, D. C.
Geo. W. Cook, Washington, D. C.
J. B. Hamilton, Washington, D. C.
C. H. A. Kleinschmidt, Washington, D. C.
Jos. M. Toner, Washington, D. C.
A. Y. P. Garnett, Washington, D. C.

FLORIDA.

N. D. Phillips, Gainesville, Fla.

GEORGIA.

J. A. Gray, Atlanta, Ga.
J. W. Bailey, Gainesville, Ga.
Thos. S. Hopkins, Louisville, Ga.
J. Mc. F. Gaston, Atlanta, Ga.
Peter R. Cortleyou, Marietta, Ga.
Thos. Sessions Dekle, Thomasville, Ga.
A. G. Whitehead, Waynesboro, Ga.
M. F. Westmoreland, Atlanta, Ga.

ILLINOIS.

Edmund Andrews, Chicago, Ill.
Philip Adolphus, Chicago, Ill.
Walter W. Allport, Chicago, Ill.
J. J. M. Angear, Chicago, Ill.
M. D. Hale, Arrowsmith, Ill.
Herman W. Alexander, Joliet, Ill.
C. C. Browning, Adrian, Ill.
A. H. Burr, Chicago, Ill.
Robt. N. Barger, Hopedale, Ill.
Robert H. Babcock, Chicago, Ill.
Frank Billings, Chicago, Ill.
J. K. Berkebile, Millstadt, Ill.
R. G. Bogue, Chicago, Ill.
John Bartlett, Chicago, Ill.
J. E. Bumsted, Dundee, Ill.
John H. Besarian, Chicago, Ill.
L. H. Baker, Payson, Ill.
B. A. Brigham, Chicago, Ill.
Jas. G. Berry, Chicago, Ill.
Norman Bridge, Chicago, Ill.
Mary E. Bates, Chicago, Ill.
James H. Broffet, Paw Paw, Ill.
Josiah Brown, Decatur, Ill.
Wm. Barritt, Onargo, Ill.
H. A. Bates, Chicago.
A. F. Burnham, Ashland, Ill.
D. R. Brower, Chicago, Ill.
J. H. Bates, Chicago, Ill.
A. E. Baldwin, Chicago, Ill.
Robert Boal, Peoria, Ill.
Boerne Bettman, Chicago, Ill.
Wm. T. Belfield, Chicago, Ill.
Truman W. Brophy, Chicago, Ill.
R. W. Crothers, Delevan, Ill.
E. P. Cook, Mendota, Ill.
Jas. M. G. Carter, Waukegan, Ill.
James Cole, Chicago, Ill.
Geo. H. Chapman, Grand Crossing, Ill.
W. S. Caldwell, Freeport, Ill.
J. E. Coleburn, Chicago, Ill.
Alex. H. Cook, Chicago, Ill.
John H. Chew, Chicago, Ill.
George L. Corcoran, Brimfield, Ill.
E. F. Cleveland, Dundee, Ill.
Wm. E. Clarke, Chicago, Ill.
Wm. J Chenoweth, Decatur, Ill.
Chas. Caldwell, Chicago, Ill.
W. E. Casselberry, Chicago, Ill.

Boyd Cornick, Mascoutah, Ill.
E. J. Doering, Chicago, Ill.
H. C. Donaldson, Morrison, Ill.
Alfred Dallberg, Chicago, Ill.
Jno. N. Dixon, Springfield, Ill.
I. E. De Olney, Chicago, Ill.
N. S. Davis, Chicago, Ill.
Isaac N. Danforth, Chicago, Ill.
Wm. Dougall, Joliet, Ill.
Nath. S. Davis, Jr., Chicago. Ill.
Fannie Dickinson, Chicago, Ill.
Charles W. Evans, Ill.
Wm. O. Ensign, Rutland, Ill.
C. W. Earle, Chicago, Ill.
J. W. Emmons, Forreston, Ill.
J. A. Freeman, Millington, Ill.
C. A. Foulks, Chicago, Ill.
Geo. F. Fiske, Chicago, Ill.
A. H. Foster, Chicago, Ill.
Corwin A. Freeman, Leland, Ill.
E. W. Fiegenbaum, Edwardsville, Ill.
Julius Fiegenbaum, Alton, Ill.
David W. Graham, Chicago, Ill.
W. M. German, Morgan Park, Ill.
Geo. H. Gilson, Shipman, Ill.
Chas. B. Goldsborough, Chicago, Ill.
Joseph L. Gray, Chicago, Ill.
Henry Gradle, Chicago, Ill.
Moses Gunn, Chicago, Ill.
A. W. Harlan, Chicago, Ill.
E. P. G. Holderness, Chenoa, Ill.
V. L. Hurlburt, Chicago, Ill.
Benj. H. Harris, Groveland, Ill.
John W. Hensley, Yates City, Ill.
J. E. Hequembourg, Chicago, Ill.
R. N. Hall, Chicago, Ill.
H. C. Holton, Sidell, Ill.
S. T. Hurst, Greenview, Ill.
J. G. Harvey, Blue Mound, Ill.
Albert E. Hoadley, Chicago, Ill.
Plymmon S. Hayes, Chicago, Ill.
H. T. Hardy, Kaneville, Ill.
S. J. Jones, Chicago, Ill.
W. W. Jaggard, Chicago, Ill.
H. C. Jones, Cerro Gordo, Ill.
E. A. Kilbourne, Elgin, Ill.
J. R. Kewley, Chicago, Ill.
E. B. Loomis, Chicago, Ill.
W. S. Linn, Bowensburg, Ill.
E. P. Murdock, Chicago, Ill.
John B. Murphy, Chicago, Ill.
Thomas M. McIlvaine, Peoria, Ill.
L. H. Montgomery, Chicago, Ill.
S. A. McWilliams, Chicago, Ill.
Jennie E. Mayner, Chicago, Ill.
Robert Roskoten, Peoria, Ill.
Geo. J. Rivard, Assumption, Ill.
Chas. G. Smith, Chicago, Ill.
J. H. Stowell, Chicago, Ill.
A. R. Small, Chicago, Ill.
I. M. Weller, Chicago, Ill.
Cassius D. Wescott, Chicago, Ill.
George W. Webster, Chicago, Ill.
Patrick Dougherty, Chicago, Ill.
C. C. P. Silva, Chicago, Ill.
Flavius M. Wilder, Chicago, Ill.
Thaddeus P. Seeley, Chicago, Ill.
Elbert Wing, Chicago, Ill.
Leslie E. Tefft, Elgin, Ill.
Sylvester Thompson, Blandinsville, Ill.
Jas. H. Wallace, Monmouth, Ill.
A. R. Van Horne, Jerseyville, Ill.
Geo. A. Zeller, Spring Bay, Ill.
Samuel N. Sims, St. Joseph, Ill.
Frank C. Shaefer, Chicago, Ill.
John H. Tyler, Clinton, Ill.
D. A. K. Steele, Chicago, Ill.
Geo. E. Willard, Chicago, Ill.
Edward B. Weston, Chicago, Ill.
Horace M. Starkey, Chicago, Ill.
Sarah H. Stevenson, Chicago, Ill.
Ira R. Wells, Geneseo, Ill.
Frank Cary, Chicago, Ill.
John T. Montgomery, Charleston, Ill.
S. S. Bishop, Chicago, Ill.
Robert Tilley, Chicago, Ill.
Frank A. Stanley, Chicago, Ill.
A. H. Taggert, Chicago, Ill.
John L. Polk, Arcola, Ill.
W. K. Sloan, Moline, Ill.
Abraham L. Thomas, Chicago, Ill.
Mary H. Thompson, Chicago, Ill.
Frank O. Stockton, Chicago, Ill.
J. F. Williams, Chicago, Ill.
G. Jackson Tobias, Chicago, Ill.
Thos. A. Scott, Galva, Ill.
Thos. W. Williams, Casey, Ill.
H. Martyn Scudder, Chicago, Ill.
Jane E. Walton, Chicago, Ill.
Henry J. B. Wright, Olney, Ill.
Edwin Swartz, Knoxville, Ill.
L. E. Spear, Shirley, Ill.
Walter L. Swan, Harrisburg, Ill.
Joseph Zeisler, Chicago, Ill.
James C. Sullivan, Cairo, Ill.
Warren M. Sweetland, Highland Park, Ill.
Frank E. Waxham, Chicago, Ill.
Jno. S. Kyburn, Ottawa, Ill.
W. H. Willis, Henry, Ill.
John F. Snyder, Monroe Centre, Ill.
John A. Robison, Chicago, Ill.
J. H. Plecker, Chicago, Ill.
H. P. Newman, Chicago, Ill.
Wm. J. Maynard, Chicago, Ill.
R. A. McClelland, Yorkville, Ill.
Catharine B. Slate, Aurora, Ill.
Edwin R. Willard, Wilmington, Ill.
K. E. Rich, Wenona, Ill.
J. J. Rendleman, Makanda, Ill.
A. J. Miller, Paris, Ill.
Christopher Goodbrake, Clinton, Ill.
Benj. F. Uran, Kankakee, Ill.
Daniel B. Collins, Chicago, Ill.
Denlow Lewis, Hyde Park, Ill.
Jno. J. Alderson, Chicago, Ill.
S. K. Crawford, Chicago, Ill.
Isaac E. Bennett, Plano, Ill.
John D. Skeer, Chicago, Ill.
John R. Webster, Monmouth, Ill.
Charity A. Sanders, Ottawa, Ill.
Jno. P. Morrison, Chicago, Ill.
Marie J. Mergler, Chicago, Ill.
Daniel C. Stillians, Chicago, Ill.
Ortho B. Will, Peoria, Ill,
John Wright, Clinton, Ill.
A. B. Strong, Chicago.
Henry Shimer, Mount Carroll, Ill.
E. S. Talbot, Chicago, Ill.
P. M. Woodworth, Chicago, Ill.
Wm. Weir Hester, Quincy, Ill.
L. P. Walbridge, Grafton, Ill.
Wm. J. Webb, Chicago, Ill.
C. C. Hunt, Dixon, Ill.
T. G. Hickman, Vandalia, Ill.

G. H. Randall, Chicago, Ill.
J. H. Hollister, Chicago, Ill.
A. B. Horam, Chicago, Ill.
J. C. Graham, Lexington, Ill.
A. Melville Tully, Chicago, Ill.
David Smith Jenks, Plano, Ill.
Henry F. Godrey, Galena, Ill.
Clar. J. Lewis, Chicago, Ill.
Erasmus Garrott, Chicago, Ill.
J. S. Knox, Chicago, Ill.
W. L. Copeland, Chicago, Ill.
Geo. W. Nesbitt, Sycamore, Ill.
C. K. Brown, Sycamore, Ill.
Ed. L. Mayo, De Kalb, Ill.
E. Ingals, Chicago, Ill.
F. S. Johnson, Chicago, Ill.
Ralph N. Isham, Chicago, Ill.
L. L. McArthur, Chicago, Ill.
J. H. Miller, Oconee, Ill.
Andrew J. McGaffigan, Carlyle, Ill.
H. P. Merriman, Chicago, Ill.
Truman W. Miller, Chicago, Ill.
Harold N. Moyer, Chicago, Ill.
Frederick W. Mercer, Chicago, Ill.
John S. Marshall, Chicago, Ill.
F. J. Parkhurst, Danvers, Ill.
Wm. L. Pollock, Heyworth, Ill.
Joseph Pogue, Edwardsville, Ill.
Malcolm G. Parsons, Carbondale, Ill
Nathan S. Marshall, Olney, Ill.
Wm. M. Richards, Joliet, Ill.
Alfred Nash, Joliet, Ill.
Orrin W. Moore, Lockport, Ill.
Robert Randolph, Chicago, Ill.
Edward L. Mitchell, Roseville, Ill.
A. V. Park, Chicago, Ill.
Chas. W. Purdy, Chicago, Ill.
S. C. Martin, Anna, Ill.
Franklin H. Martin, Chicago, Ill.
M. M. Robbins, Aurora, Ill.
Charles T. Parkes, Chicago, Ill.
Daniel T. Nelson, Chicago, Ill.
John H. Mitchell, Mt. Vernon, Ill.
Edwin Pynchon, Chicago, Ill.
Chas. E. Winslow, Aurora, Ill.
Geo. W. Mason, Bloomington, Ill.
Lee Smith, Bloomington Ill.
George Wheeler Jones, Danville, Ill.
M. T. Gamble, Farmington, Ill.
Chas. S. Laughlin, Paris, Ill.
A. W. Kinnear, Henry, Ill.
G. Frank Lydston, Chicago, Ill.
W. T. Montgomery, Chicago, Ill.
Arthur R. Reynolds, Chicago, Ill.
John E. Owens, Chicago, Ill.
Charles P. Pruyn, Chicago, Ill.
J. E. Rhodes, Chicago, Ill.
Henry J. Reynolds, Chicago Ill.
DeLaskie Miller, Chicago, Ill.
R. J. Patterson, Batavia, Ill.
T. J. Pitner, Jacksonville, Ill.
Edwin Powell, Chicago, Ill.
Wm. Gardner Putney, Prairie Centre, Ill.
Sam. C. Plummer, Rock Island, Ill.
Sam. C. Plummer, Jr., Rock Island, Ill.
W. W. McMann, Gardner, Ill.
Eliza H. Root, Chicago, Ill.
Fred L. Nutt, Marengo, Ill.
J. M. Hall, Chicago, Ill.
Ellen Costin Gage, Winnetka, Ill.
Ezra T. Goble, Earlville, Ill.
Hosmer A. Johnson, Chicago. Ill.
R. L. Leonard, Chicago, Ill
D. W. Jump, Plainfield, Ill.
Helen R. Kellogg, Chicago, Ill.
Walter Hay, Chicago, Ill.
John E. Harper, Chicago, Ill.
H. H. Frothingham, Chicago, Ill.
Christian Fenger, Chicago, Ill.
E. F. Ingals, Chicago, Ill.
Jos. W. Wassall, Chicago, Ill.
W. L. Downey, Wenona, Ill.
John Little, Bloomington, Ill.
Wm. S. Holliday, Monmouth, Ill.
A. J. C. Saunier, Libertyville, Ill.
Wm. H. Fitch, Rockford, Ill.
Jno. R. McCullough, Chicago, Ill.
J. E. Charles, Peoria, Ill.
E. H. Keith, Peoria, Ill.
A. Reeves Jackson, Chicago, Ill.
John W. Green, Marengo, Ill.
Jacob S. Kaufman, Blue Island, Ill.
S. M. Sawyers, Unionville, Ill.
Henry B. Brown, Lincoln, Ill.
G. M. Chamberlain, Chicago, Ill.
W. B. Stiver, Stephenson, Ill.
H. H. Denning, Pana, Ill.
Ira N. Barnes, Decatur, Ill.
Wm. M. Harsha, Decatur, Ill.
Oscar C. DeWolf, Chicago, Ill.
Chas. P. Parke, Bloomington, Ill.
Robert Hughes, Okawville, Ill.
John T. Hopkins, Chicago, Ill.
Mary C. King, Aurora, Ill.
Louis Nathan Barlow, Chicago, Ill.
T. C. Cullen, Chicago, Ill.
Jno. C. Cook, Hyde Park, Ill.
Geo. G. Parker, Cairo, Ill.
Malcolm T. Moore, Dunning, Ill.
Jno. E. Best, Abingdon Heights, Ill.
Walter S. Haines, Chicago, Ill.
Rosa H. Engert, Chicago.
Jno. B. Hench, Hinsdale, Ill,
D. E. Burlingame, Elgin, Ill.
Wm. T. Akins, Chicago.
Robert S. Wall, Chicago.
T. D. Fitch, Chicago.
J. T. Pearman, Champaign, Ill.
Wm. A. Cole, Jacksonville, Ill.
Ferdinand C. Holz, Chicago.
Robert J. Mitchell, Girard, Ill.
J. L. Million, Springfield, Ill.

INDIANA.

Geo. W. Crapo, Terre Haute, Ind.
L. L. Todd, Indianapolis, Ind.
John A. Sutcliffe, Indianapolis, Ind.
Ervin Wright, Roanoke, Ind.
J. C. Sexton, Rushville, Ind.
Geo. W. Welborn, Stewartsville, Ind.
Salathiel T. Williams, Kendallville, Ind.
G. Theo. Bourbach, Ft. Wayne, Ind.
G. V. Woolen, Indianapolis, Ind.
F. M. Pearman, Palestine, Ind.
Allen Piersen, Spencer, Ind.
Ezra B. Evans, Greencastle, Ind.
David R. Walker, Reese's Mill, Ind.
C. B. Miller, Lawrenceburgh, Ind.
Wm. H. Shulse, Lebanon, Ind.
Edward G. White, La Grange, Ind.
B. S. Woodworth, Ft. Wayne, Ind.
Jacob R. Weist, Richmond, Ind.
James L. Thompson, Indianapolis, Ind.
G. W. Thompson, Winamac, Ind.

Carter H. Smith, Lebanon, Ind.
John H. Spurrier, Rushville, Ind.
A. B. Younkman, Bremen, Ind.
Willam Burton Thomas, Fairmount, Ind.
Sheton H. Shaptaugh, Princeton, Ind.
Calvin L. Shull, Montpelier, Ind.
Jas. H. Whitcomb, Boswell, Ind.
Wm. H. Short, La Grange, Ind.
T. J. Schackleford, Warsaw, Ind.
Philip P. Whitesell, Clarksville, Ind.
Wm. V. Wiles, Spencer, Ind.
John S. Sproul, Warren, Ind.
Wm. S. Walker, LaFayette, Ind.
H. C. Vincent, Guilford, Ind.
Richard B. Wetherill, LaFayette, Ind.
Chas. H. Parsons, Rushville, Ind.
La Grange Severaine, Huntington, Ind.
James Anderson Work, Elkhart, Ind.
Samuel C. Thomas, Milroy, Ind.
J. H. Wilson, Plymouth, Ind.
J. V. Schofield, Harris City, Ind.
Geo. W. Shepherd, Red Key, Ind.
John B. Weaver, Evansville, Ind.
Luther D. Waterman, Indianapolis, Ind.
Lewis Williams, Marion, Ind.
John Rhea, New Castle, Ind.
Robt. S. Rutherford, Galena, Ind.
F. D. Norton, Petersville, Ind.
Charles W. McIntyre, New Albany, Ind.
Benjamin Newland, Bedford, Ind.
John Z. Powell, Logansport, Ind.
Thos. E. Powell, Evansville, Ind.
John E. Owen, Evansville, Ind.
James. A. Meek, Bunker Hill, Ind.
Isaac N. Rosenthal, Fort Wayne, Ind.
W. A. Neal, Elkhart, Ind.
Delos D. Marr, Chesterton, Ind.
A. C. Holtzendorff, Plymouth, Ind.
John E. Link, Terre Haute, Ind.
Thos. B. Mooney, Indianapolis, Ind.
B. H. B. Grayston, Huntington, Ind.
C. B. Wiggins, Peru, Ind.
E. F. Hodges, Indianapolis, Ind.
R. E. Miller, Chesterton, Ind.
I. T. Linn, Bourbon, Ind.
Jas. R. Hinkle, Sullivan, Ind.
S. W. Lemmon, Albion, Ind.
Edwin R. Lewis, Crawfordsville, Ind.
L. M. Irwin, Lafayette, Ind.
F. S. C. Graystone, Huntington, Ind.
J. T. McShane, Carmel, Ind.
Jacob W. Eidson, Bourbon, Ind.
Frank C. Ferguson, Indianapolis, Ind.
Geo. J. Cook, Indianapolis, Ind.
Wm. B. Spencer, Terre Haute, Ind.
Geo. H. La Grange, Dayton, Ind.
Samuel S. Home, Jonesboro, Ind.
Eugene B. North, Peru, Ind.
J. J. Becknell, Milford, Ind.
G. W. Arnold, Valparaiso, Ind.
R. H. Culbertson, Brazil, Ind.
Chas. P. Cook, New Albany, Ind.
Geo. F. Beasley, Lafayette, Ind.
S. M. Cunius, Elkhart, Ind.
Marc. L. Bond, Aurora, Ind.
A. B. Dearby, Waterloo, Ind.
John Chambers, Indianapolis, Ind.
C. W. Burkett, Warsaw, Ind.
Wm. Flynn, Marion, Ind.
L. H. Dunning, South Bend, Ind.
John C. Fulton, Murray, Ind.
W. T. Lawson, Danville, Ind.
John M. Neeler, Peru, Ind.
J. P. Henderson, Salem, Ind.
John R. Dacer, South Milford, Ind.
John M. Bash, Warsaw, Ind.
Wm. C. Chaffee, Huntingdon, Ind.
Chas. D. Bond, Richmond, Ind.
F. W. Bearde, Vincennes, Ind.
Geo. W. Burke, Newcastle, Ind.
Geo. S. Jones, Covington, Ind.
Jas. R. Adams, Petersburg, Ind.
James F. Hibberd, Richmond, Ind.
William C. Davis, Indianapolis, Ind.
Rufus R. Kime, Petersburg, Ind.
C. I. Fletcher, Indianapolis, Ind.
G. W. Bucklin, New Harmony, Ind.
Clark Cook, Fowler, Ind.
Nelson Chenowith, Windsor, Ind.
Lewis Davis, Farmland, Ind.
D. B. Armitage, Muncie, Ind.
John Chenowith, Winchester, Ind.
M. H. Bonnell, Lebanon, Ind.
Joseph Eastman, Indianapolis, Ind.
Thos. W. Farshee, Madison, Ind.
Alphus Henley, Fairmont, Ind.
Jas. B. Greene, Mishawaka, Ind.
John B. Hozel, Clay Pool, Ind.
Thos. M. Love, Lebanon, Ind.
S. V. Jessup, New Burlington, Ind.
A. M. Good, Selma, Ind.
Wm. C. Henry, Aurora, Ind.
V. H. Gregg, Connersville, Ind.
Wm. M. Holton, New Harmony Ind.
J. H. Wm. Meyer, La Porte, Ind.
Ed. T. Hodges, Indianapolis, Ind.
Ephraim A. Rogers, La Porte, Ind.
George W. McCaskey, Ft. Wayne, Ind.
Homer C. Marklane, Independence, Ind.
Chas. C. Dougherty, South Bend, Ind.
A. J. Banker, Columbus, Ind.
Landon C. Rose, La Porte, Ind.
Wm. B. Fletcher, Indianapolis.
Chas. M. Fry, Brochen, Ind.
Wm. R. Moffitt, Lafayette, Ind.
Toliver Weitz, Jasper, Ind.
Wm. R. McMahon, Huntingburgh, Ind.
G. P. Williams, Huntingburgh, Ind.
J. A. Baker, Stockwell, Ind.
J. L. P. Ward, Union, Ind.
A. M. Hayden, Evansville, Ind.
Lorenzo S. Keene, La Porte, Ind.
John C. Webster, La Fayette, Ind.
P. M. O'Terral, South Lafayette, Ind.
Frank M. Sawyer, South Bend, Ind.
Benj. T. Freeman, Decatur, Ind.

IOWA.

W. S. Grimes, Wapello, Iowa.
J. W. Holliday, Burlington, Iowa.
John B. Ingels, Meriden, Iowa.
Chas. B. Powell, Albia, Iowa.
B. Erp Brockhausen, Lansing. Iowa.
David S. Fairchild, Ames, Iowa.
T. J. Maxwell, Keokuk, Iowa.
W. D. Campbell, Marshalltown, Iowa.
A. K. Berry, Chillicothe, Iowa.
G. F. Jenkins, Keokuk, Iowa.
E. M. Gould, Des Moines, Iowa.
R. A. Patchin, Des Moines, Iowa.
A. M. Vail, Rock Rapids, Iowa.
Daniel C. Greenleaf, Bloomfield, Iowa.
F. C. Jones, Herndon, Iowa.
J. C. Joralemon, Toledo, Iowa.

Wm. Gutch, Albia, Iowa.
David B. Hillis, Keokuk, Iowa.
T. B. McWilliams, Sigourney, Iowa.
W. F. Peck, Davenport, Iowa.
Sanford T. Goodman, Maxwell Iowa.
William Fitzgerald, Grand Mound, Iowa.
H. S. Hadsel, Maynard, Iowa.
Elliot Cobb, Harlan, Iowa.
J. Craig Dunlavy, Harlan, Iowa.
R. A. Dunkelberg, Denver, Iowa.
Chas. Enfield, Jefferson, Iowa.
L. L. Bond, West Side, Iowa.
A. O. Williams, Ottumwa, Iowa.
Galloway Trunx, Marquette, Iowa.
J. P. Crawford, Davenport, Iowa.
Gilbert Baldwin, Ruthven, Iowa.
Edwin Blakeslee, Anamosa, Iowa.
Andrew J. Hobart, Clinton, Iowa.
W. L. Duffin, Guttenberg, Iowa.
Geo. W. Beggs, Sioux City, Iowa.
Thos. J. Schnell, Parnell, Iowa.
Walter B. Sherman, Manchester, Iowa.
Elizabeth Hess, Iowa City, Iowa.
Elmer F. Clapp, Iowa City, Iowa.
Horace B. Ransom, Burlington, Iowa.
J. W. Philpott, Ft. Madison, Iowa.
J. S. Roome, Calmar, Iowa.
F. W. Porterfield, Atlantic, Iowa.
Andrew W. McClure, Mt. Pleasant, Iowa.
W. D. Middleton, Davenport, Iowa.
F. C. Mehler, New London, Iowa.
J. W. Young, Bloomfield, Iowa.
Edgar J. Meacham, Washington, Iowa.
John M. Ristine, Cedar Rapids, Iowa.
Frank A. Weir, Jessup, Iowa.
D. McTavish, Central City, Iowa.
John M. Sherman, Paton, Iowa.
Robert R. Williams, Manning, Iowa.
Lewis Schooler, Des Moines, Iowa.
Arthur Len Wright, Carroll, Iowa.
Fred. M. Ward, Marshalltown, Iowa.
F. R. Smith, Pleasant Plain, Iowa.
Griffin B. Ward, Fairbank, Iowa.
Wm. Van Velsor, Renwick, Iowa.
Jno. L. Sawyer, Centreville, Iowa.
John H. Thornton, Lansing, Iowa.
Saml. Thompson, Toledo, Iowa.
D. H. Worthington, Fairfield. Iowa.
J. A. Scroggs, Keokuk, Iowa.
Henry B. Young, Burlington, Iowa.
Darius Schofield, Washington, Iowa.
John E. Wilkinson, Ottumwa, Iowa.
Fred J. Will, Eagle Grove, Iowa.
Joel W. Smith, Charles City, Iowa.
Milton G. Sloan, Dexter, Iowa.
M. W. White, Van Horne, Iowa.
Wm. Watson, Dubuque, Iowa.
Jas. A. Sherman, Cherokee, Iowa.
Millard M. Newman, Edgewood, Iowa.
Benjamin McCluer, Dubuque, Iowa.
D. W. Overholt, Columbus Junction, Iowa.
Ed. B. Plumb, Ames, Iowa.
Herbert Marcus McKenzie, Elwood, Iowa.
Albert Reynolds, Clinton, Iowa.
C. H. Philpott, Albia, Iowa.
W. F. S. Murdy, Moulton, Iowa.
J. H. Nicol, Lacona, Iowa.
Walter O. Richards, Waterloo, Iowa.
Samuel N. Pierce, Cedar Falls, Iowa.
Stella B. Nichols, Davenport, Iowa.
Wm. H. McLain, Bauman, Iowa.
C. S. Paul, Paullina, Iowa.
H. Ristine, Cedar Rapids, Iowa.
A. B. Reed, Cedar Rapids, Iowa.
Milton I. Powers, Parkersburgh, Iowa.
H. R. Page, Des Moines, Iowa.
John North, Keokuk, Iowa.
N. J. A. Mueler, New Vienna, Iowa.
H. C. McCleary, Indianola, Iowa.
Allen A. Rawson, Corning Iowa.
Jas. D. Reynolds, Creston, Iowa.
Jas. B. Wilson, Creston, Iowa.
Wm. H. Davies, Maquoketa, Iowa.
Isaiah Merrills Harsh, Cumberland, Iowa.
O. E. Evans, Gowrie, Iowa.
M. Lander Petz, Marshalltown, Iowa.
G. E. Crawford, Cedar Rapids, Iowa.
Daniel W. Crouse, Waterloo, Iowa.
Luther Brown, Postville, Iowa.
Samuel D. Cook, Sigourney, Iowa.
W. S. Brainard, Stacyville, Iowa.
Azro Bundley, St. Ansgar, Iowa.
John M. Auld, Keota, Iowa.
O. W. Lowery, Grand Junction, Iowa.
C. G. Lewis, Ottumwa, Iowa.
G. H. Hill, Independence, Iowa.
J. H. Lyon, Moingona, Iowa.
H. A. Gilman, Mt. Pleasant, Iowa.
Lyman J. Adair, Anamosa, Iowa.
Ed. E. Kirkendall, West Burlington, Iowa.
John P. Kaster, Burlington, Iowa.
T. J. Douglass, Ottumwa, Iowa.
John A. Riggan, What Cheer, Iowa.
P. W. Lewellen, Clarinda, Iowa.
Norman L. Kean, Northwood, Iowa.
Leonard R. Garfield, Algona, Iowa.
Chanley J. Lukens, New Sharon, Iowa.
Woods Hutchinson, Des Moines, Iowa.
J. S. Long, Springville, Iowa.
E. H. King, West Liberty, Iowa.
Rollo J. Most, Low Moor, Iowa.
Jos. C. Hinsey, Ottumwa, Iowa.
E. A. Kegley, Cedar Rapids, Iowa.
C. M. Hobby, Iowa City, Iowa.
Jos. C. Hughes, Keokuk, Iowa.
S. B. Chase, Osage, Iowa.
J. T. Priestley, Des Moines, Iowa.
W. H. Ward, Des Moines, Iowa.
Louis Blanchard, Edgewood, Iowa.
A. A. Deering, Boone, Iowa.
Norman A. Craig, Manchester, Iowa.
W. C. Egan, Atlantic, Iowa.
Geo. P. Hanawalt, Des Moines, Iowa.
B. H. Criley, Dallas Center, Iowa.
B. G. Kimmel, Winfield, Iowa.
Henry W. Brown, Waterloo, Iowa.
Nancy M. Hill, Dubuque, Iowa.
W. T. Eckley, Harper, Iowa.
Jas. A. Treat, Stewart, Iowa.
Robt. U. Payner, Richland, Iowa.
Chas. D. Moore, Des Moines, Iowa.
Jennie McCowan, Davenport, Iowa.
W. C. Eselbock, Iowa.
C. E. Currie, De Moines, Iowa.
David W. Loose, Iowa.
Henry M. Mixer, New Hampton, Iowa.
Samuel S. Lytle, Iowa City, Iowa.
G. W. H. Baxter, Willow Junction, Iowa.
W. W. Grant, Davenport, Iowa.

KANSAS.

W. A. McCully, Independence, Kan.
J. E. Oldham, Wichita, Kan.

William Addison Philips, Salina, Kan.
John Troutman, Wyandotte, Kan.
Herbert K. Tifft, Topeka, Kan.
James Bell, Olathe, Kan.
John E. Minney, Topeka, Kan.
W. L. Schenck, Osage, Kan.
D. W. Stormont, Topeka, Kan.
Levi Hornor, Lawrence, Kan.
Deboss K. Longsley, Topeka, Kan.
G. R. Baldwin, Fort Scott, Kan.
Robt. Aikman, Fort Scott, Kan.
A. H. Fabrique, Wichita, Kan.

KENTUCKY.

Jas. N. Letcher, Henderson. Ky.
Geo. W. Griffiths, Louisville, Ky.
Wm. Cheatham, Louisville, Ky.
Wm. M. Hanna, Henderson, Ky.
Wm. Bailey, Louisville, Ky.
Wm. H. Wathen, Louisville, Ky.
F. Dunlap, Danville, Ky.
W. A. Jordan, Clinton, Ky.
S. W. Luten, Cayce, Ky.
John A. Larrabee, Louisville, Ky.
John Y. Brooks, Paducah, Ky.
J. B. Bailey, Olmstead, Ky.
H. Brown, Houstonville, Ky.
Geo. Beele, Clinton, Ky.
Dudley S. Reynolds, Louisville, Ky.
R. C. McChord, Lebanon, Ky.
W. W. Richmond, Clinton, Ky.
P. W. McKeel, Wingo, Ky.
W. O. Roberts, Louisville, Ky.
M. F. Coomes, Louisville, Ky.
John Godfrey, Louisville, Ky.

LOUISIANA.

Roberdean W. Seay, Pilcher's Point, La.
T. G. Richardson, New Orleans, La.

MASSACHUSETTS.

Arthur H. Wilson, S. Boston, Mass.
Grace Wolcott, Boston, Mass.
F. K. Paddock, Pittsfield, Mass.
Levi F. Warner, Boston, Mass.
E. W. Cushing, Boston, Mass.
Robt. F. Andrews, Gardner, Mass.
Chas. B. Belt, Boston, Mass.
J. W. Johnson, Boston, Mass.
Henry O. Marcy, Boston, Mass.
M. G. Parker, Lowell, Mass.

MAINE.

Thos. A. Foster, Portland, Maine.
Alonzo Garcelon, Lewiston, Me.
D. E. Marston, Monmouth, Maine.
H. P. Merrill, Portland, Me.

MARYLAND.

John Morris, Baltimore, Md.
Geo. H. Rohe, Baltimore, Md.
E. M. Reid, Baltimore, Md.
L. D. Gorgas, Baltimore, Md.
J. S. Lynch, Baltimore, Md.
A. B. Arnold, Baltimore, Md.
Julian J. Chisholm, Baltimore, Md.
Thomas B. Evans, Baltimore, Md.

MICHIGAN.

Theo. A. McGraw, Detroit, Mich.
Peter E. Richmond, Mt. Pleasant, Mich.
John J. Mulheron, Wayne, Mich.
George E. Ranney, Lansing, Mich.
Isaac E. Randall, West Bay City, Mich.
Charles Bliss Stockwell, Port Huron, Mich.
Walter E. Ward, Laingsburg, Mich.
Lewis E. Main, Detroit, Mich.
Horace Tupper, Bay City, Mich.
C. V. Tyler, Bay City, Mich.
Leander D. Tompkins, Cassopolis, Mich.
H. C. Wyman, Detroit, Mich.
D. McLeay, Prairieville, Mich.
D. W. C. Wade, Holly, Mich.
Clarence H. White, Reed City, Mich.
O. M. Vaughn, Covert, Mich.
Thos. J. Sullivan, Ann Arbor, Mich.
Oscar F. Seeley, Climax, Mich.
Carter S. Van Antwerp, Vicksburg, Mich.
John Vander Laan, Muskegon, Mich.
Ezra A. Palmer, Hartford, Mich.
Gilbert L. Rose, Decatur, Mich.
Fred. W. Mann, Detroit, Mich.
Wm. J. McHench, Brighton, Mich.
Samuel V. Romig, Ionia, Mich.
Donald Maclean, Detroit, Mich.
Herman H. Schaber, Kalamazoo City, Mich.
Robert Stephenson, Adrian, Mich.
Irwin Simpson, Kalamazoo, Mich.
David H. Wood, Quincy, Mich.
Victor C. Vaughan, Ann Arbor, Mich.
Chas. W. Morse, Dowagiac, Mich.
A. B. Palmer, Ann Arbor, Mich.
J. L. Burkart, Big Rapids, Mich.
John Bell, Benton Harbor, Mich.
Jas. M. Cook, Muskegon, Mich.
E. B. Chapin, Grass Lake, Mich.
J. N. Buckham, Flint, Mich.
J. H. Carstens, Detroit, Mich.
Geo. O. Hilton, Paw Paw, Mich.
Geo. H. Lowry, Hastings, Mich.
D. E. Fuller, Nashville, Mich.
Alonzo Garwood, Cassopolis, Mich.
S. Belknap, Niles, Mich.
Helen W. M. Kirkland, Kalamazoo, Mich.
Wm. J. Kelsey, Cassopolis, Mich.
C. H. Lewis, Jackson, Mich.
Charles Shepard, Grand Rapids, Mich.
E. B. Dunning, Paw Paw, Mich.
Henry B. Baker, Lansing, Mich.
W. H. Landis, Woodland, Mich.
Wm. Brodie, Detroit, Mich.
Simon French, Battle Creek, Mich.
A. W. Alvord, Battle Creek, Mich.
C. C. Dillenbaugh, Portland, Mich.
Lyman W. Bliss, Saginaw, Mich.
B. Buckham, Flint, Mich.
Hugh McColl, Lapeer, Mich.
Geo. E. Frothingham, Ann Arbor, Mich.
Amy Garrison Kinball, Jackson, Mich.
H. O. Walker, Detroit, Mich.
Chas. J. Lundy, Detroit, Mich.
P. A. Knight, Metica, Mich.
Wm. Geo. Hervey, Detroit, Mich.
Robert Le Baron, Pontiac, Mich.
J. T. Jenkins, Tecumseh, Mich.
Jos. Groner, Big Rapids, Mich.
Leartus Connor, Detroit, Mich.
Carl Brumme, Detroit, Mich.
I. E. Brown, Detroit, Mich.
Amos Knight, Eaton Rapids, Mich.
Hiram Dessinger, Canal Fulton, Mich.
H. B. Hemenway, Kalamazoo, Mich.
Jno. H. Kellogg, Battle Creek, Mich.

J. E. Eanerson, Detroit, Mich.
Philo B. Patterson, Charlotte, Mich.

MINNESOTA.

J. Warren Little, Minneapolis, Minn.
John H. Murphy, St. Paul, Minn.
Frederick A. Dunsmoor, Minneapolis, Minn.
Geo. F. French, Minneapolis, Minn.
Chas. N. Hewitt, Red Wing, Minn.
Horace H. Wilberstine, Rochester, Minn.
Cornelius Williams, St. Paul, Minn.
Franklin Staples, Winona, Minn.
James Fulton Percy, Mazeppa, Minn.
N. S. Lane, Eyota, Minn.
A. McLarin, St. Paul, Minn.
Park Ritchie, St. Paul, Minn.
Justus Ohage, St. Paul, Minn.
Chas. E. Rogers, Montevideo, Minn.
William W. Mayo, Rochester, Minn.
J. B. McGaughey, Winona, Minn.
A. W. Stinchfield, Eyota, Minn.
J. H. Stewart, St. Paul, Minn.
A. A. Gilman, St. Cloud, Minn.
E. W. Cross, Rochester, Minn.
H. L. Coon, Northfield, Minn.
P. H. Millard, Stillwater, Minn.
F. J. Hutton, Fergus Falls, Minn.
Chas. Hill, Pine Island, Minn.
Edgar B. Holmes, Oronoco, Minn.
H. P. Johnson, Houston, Minn.
Alex. J. Stone, St. Paul, Minn.

MISSISSIPPI.

Robt. B. Carson, Durant, Miss.

MISSOURI.

Chas. P. Cathcart, Kansas City, Mo.
L. H. Laidey, St. Louis, Mo.
Geo. R. Hughsmith, DeWitt, Mo.
Edward Borck, St. Louis, Mo.
H. O. Leonard, Kansas City, Mo.
Avis Smith, Kansas City, Mo.
Pinckney French, Mexico, Mo.
Thos. F. Rumbold, St. Louis, Mo.
A. H. Ohmann-Dumesnil, St. Louis, Mo.
Jas. E. Logan, Kansas City, Mo.
A. W. Graham, Holstein, Mo.
Dora Greene, Chillicothe, Mo.
B. G. Dysart, Paris, Mo.
N. C. Dalton, St. Louis, Mo.
Chas. P. Goodrich, New Haven, Mo.
J. H. Duncan, Kansas City, Mo.
Frank R. Fry, St. Louis, Mo.
Marcus A. Bogie, Kansas City, Mo.
John M. Allen, Liberty, Mo.
John O. F. Delaney, St. Louis, Mo.
J. H. McKellops, St. Louis, Mo.
Frank J. Lutz, St. Louis, Mo.
Henry Hickman, St. Louis, Mo.
R. M. Jordan, St. Louis, Mo.
R. J. Hill, St. Louis, Mo.
Nicholas Guhman, St. Louis, Mo.
H. W. Latham, Lathamston, Mo.
David C. Gore, Paris, Mo.
Randal R. Hunter, Kansas City, Mo.
Eugene R. Lewis, Kansas City, Mo.
C. Lester Hall, Marshall, Mo.
Charles H. Lester, Kansas City, Mo.
Francis M. Johnson, Kansas City, Mo.
F. A. Glasgow, St. Louis, Mo.
W. C. Glasgow, St. Louis, Mo.
R. T. Henderson, Shawneetown, Mo.
E. H. Gregory, St. Louis, Mo.
L. Ch. Boisliniere, St. Louis, Mo.
Lyman A. Berger, Kansas City, Mo.
John T. Eggers, Kansas City, Mo.
Chas. W. Adams, Kansas City, Mo.
Wm. W. Dougherty, Liberty, Mo.
A. M. Crow, Kansas City, Mo.
George Halley, Kansas City, Mo.
Joel Reiger, Kansas City, Mo.
Isaac N. Miller, Mound City, Mo.
Eustachius Clearville, St. Louis, Mo.
J. L. Burke, La Clede, Mo.
Ed. Neville Chastain, Hume, Mo.
S. Pollak, St. Louis, Mo.
I. N. Love, St. Louis, Mo.
E. A. Waggoner, Carrollton, Mo.
A. H. Fuller, St. Louis, Mo.
P. S. O'Riley, St. Louis, Mo.
A. H. Meisenbach, St. Louis, Mo.
Frank M. Rumbold, St. Louis, Mo.
H. H. Middlekamp, Warrenton, Mo.
Alex. J. Mullen, St. Louis Mo.
Wm. M. Brown, Lewiston, Mo.
L. T. Standly, La Clede, Mo.
J. B. Wood, Marshall, Mo.
Chas. A. Thompson, Jefferson City, Mo.
J. Henry Thompson, Kansas City, Mo.
Edw. Schauffler, Kansas City, Mo.
A. B. Sloan, Kansas City, Mo.
Willard P. Sexton, New Bloomfield, Mo.
Flavel B. Tiffany, Kansas City, Mo.
A. H. Scott, St. Joseph, Mo.
Wesley Humphrey, Kansas City, Mo.
John H. Van Emmons, Kansas City, Mo.
J. M. Scott, St. Louis, Mo.
Wm. Porter, St. Louis, Mo.
John J. Miller, St. Louis, Mo.
Gratz A. Moses, St. Louis, Mo.
A. B. Carson, St. Louis, Mo.
P. S. O'Reilley, St. Louis, Mo.
C. B. Shaw, St. Louis, Mo.

NEBRASKA.

Richard C. Moore, Omaha, Neb.
John H. Quinn, Blue Springs, Neb.
Wm. M. Knapp, Lincoln, Neb.
Alex. S. Mansfield, Ashland, Neb.
A. F. Jonas, Omaha, Neb.
V. H. Coffman, Omaha, Neb.
J. E. Summers, Jr., Omaha, Neb.
W. P. Wilcox, Omaha, Neb.
E. W. Lee, Omaha, Neb.
N. B. Larsh, Nebraska City, Neb.
Alice Emily Huff, Lincoln, Neb.
Thos. P. Livingston, Plattsmouth, Neb.
G. W. Johnston, Fairmont, Neb.
Joseph T. Steele, Hastings, Neb.
G. H. Peebles, David City, Neb.
Joseph Neville, Omaha, Neb.
John P. Lord, Omaha, Neb.
Ewing Brown, Omaha, Neb.

NEW HAMPSHIRE

Winslow B. Porter, Walpole, N. H.
John W. Parsons, Portsmouth, N. H.

NEW MEXICO.

Russell Bayley, Las Vegas, New Mex.

NEW JERSEY

L. H. Southward, Newark, N. J.
Chas. Vogler, Newark, N. J.
D. W. Smith, Newark, N. J.

J. Wadsworth Terry, Englewood, N. J.
Wm. J. Swinton, Somerville, N. J.
Anson A. Ransom, South Orange, N. J.
Rich. E. Brown, Mt. Holly, N. J.
Isaac N. Quimby, Jersey City, N. J.
H. G. Buckingham, Clayton, N. J.
Ed. J. Ill, Newark, N. J.
L. S. Hinkley, Newark, N. J.
B. H. Watson, Jersey City, N. J.

NEW YORK.

Ed. M. Moore, Rochester, N. Y.
Van de Warker Ely, Syracuse, N. Y.
Henry C. Sutton, Rome, N. Y.
G. R. Martine, Glen's Falls, N. Y.
James R. Taylor, New York City, N. Y.
C. C. Wyckoff, Buffalo, N. Y.
Jno. P. Sharer, Little Falls, N. Y.
Henry Ernst Schmidt, White Plains, N. Y.
J. Lewis Smith, New York, N. Y.
Edward Charles Spilzka, New York City, N. Y.
Reginald H. Sayre, New York, N. Y.
A. R. Robinson, New York, N. Y.
Geo. W. Earle, Tully, New York.
Frank Kenyon, Scipio, N. Y.
J. C. Hannon, Hoosac Falls, N. Y.
Henry W. Carpenter, Oneida, N. Y.
Darwin Colvin, Clyde, N. Y.
J. H. H. Burge, Brooklyn, N. Y.
L. Duncan Bulkley, New York City.
A. G. Hull, New York City, N. Y.
W. W. Potter, Buffalo, N. Y.
Robert Newman, New York, N. Y.
C. O. Jackson, Victor, N. Y.
Walter Wyman, New York, N. Y.
J. B. Andrews, Buffalo, N. Y.
Chas. W. Brown, Elmira, N. Y.
A. N. Bell, Brooklyn, N. Y.
James Edward Kelley, New York.

NORTH CAROLINA.

Chas. F. McGahan, Ashvillle, N. C.
John H. Tucker, Henderson, N. C.
Eugene Grisson, Raleigh, N. C.
Chas. Jas. O'Hagan, Greenville, N. C.

OHIO.

H. J. Donahoe, Sandusky, Ohio.
H. G. Hart, Wooster, Ohio.
Salmon Hudson, Medina, Ohio.
Wm. C. Frew, Coshocton, Ohio.
Chas. Graefe, Sandusky, Ohio.
A. H. Brundage, Xenia, Ohio.
A. C. Belden, Akron, Ohio.
Horatio A. Hamilton, Perrysburg, Ohio.
F. D. Baine, Kenton, Ohio.
J. Snodgrass, Kenton, Ohio.
B. F. Clark, Cincinnati, Ohio.
Alex. Dunlap, Springfield, Ohio.
Geo. A. Callamore, Toledo, Ohio.
Wm. A. Focht, New Riegel, Ohio.
C. G. Comegys, Cincinnati, Ohio.
W. W. Dawson, Cincinnati, Ohio.
O. W. Alcord, Ravenna, Ohio.
O. N. Weise, Cincinnati, Ohio.
Norman Gay, Columbus, O.
F. Gunsaulus, Columbus, O.
F. S. Jones, Medina, Ohio.
C. C. Hoover, Ross, O.
Sol B. Hisner, Lima, O.
J. F. Gabriel, Piqua, O.
John Cass, Hamilton, Ohio.
Wm. H. Bunce, Oberlin, Ohio.
Dudley Allen, Oberlin, Ohio.
A. H. Stephens, Eaton, Ohio.
Geo. F. Cook, Oxford, Ohio.
H. J. Herrick, Cleveland, Ohio.
W. C. Jacobs, Akron, Ohio.
Edmund C. Carr, Coshocton, Ohio.
Jonathan Morris, Ironton, Ohio.
J. C. Mortland, Edgerton, Ohio.
Albert G. Miner, Niles, Ohio.
E. J. McCollum, Tiffin, Ohio.
P. P. Pomerene, Berlin, Ohio.
William Taft, Cincinnati, Ohio.
John E. Woodbridge, Youngstown, Ohio.
Julian Harman, Warren, Ohio.
Anson R. Smart, Toledo, Ohio.
H. J. Sharp, London, O.
Jonathan Taft, Cincinnati, Ohio,
David Watson, Bellefontaine, Ohio.
Jonathan B. Vail, Lima, Ohio,
Xenophon C. Scott, Cleveland, Ohio.
Cornelius N. Lyman, Wadsworth, Ohio.
John H. Tressel, Alliance, Ohio.
Byron Stanton, Cincinnati, Ohio.
Lorin W. Pontius, Canton, Ohio.
Akin C. Miller, Cleveland, Ohio.
C. C. McLaughlin, Dunkirk, Ohio.
Ben. R. McClellan, Xenia, Ohio.
C. R. Reed, Middleport, Ohio.
W. W. Firestone, Wooster, Ohio.
W. S. Powell, Defiance, Ohio.
A. H. McCullough, Mansfield, Ohio.
Aug. J. Garone, Sandusky, Ohio.
Jno. E. Russell, Mt. Vernon, Ohio.
Frank C. Larimore, Mt. Vernon, Ohio.
John K. Woods, Van Wert, Ohio.
Carl H. Von Klein, Dayton, O.
Jno. W. Wright, Columbus, Ohio.
Chas. B. Parker, Cleveland, Ohio.
I. W. Hamilton, Columbus, Ohio.

PENNSYLNANIA.

John M. Batten, Pittsburgh, Penn.
Geo. B. Dunmire, Philadelphia, Pa.
E. P. Allen, Athens, Pa.
Rich. J. Dunglison, Philadelphia, Pa.
Thos. M. Drysdale, Philadelphia, Pa.
J. J. Buchanan, Pittsburgh, Pa.
J. E. Baker, Lancaster, Pa.
D. C. Brandes, Erie, Pa.
E. L. W. Mosbourg, Johnstown, Pa.
D. E. Hughes, Williamsport. Pa.
Edward Jackson, Philadelphia, Pa.
R. B. Ewing, West Grove, Pa.
T. Ridgeway Barker, Philadelphia, Pa.
Chas R. Earley, Ridgeway, Penn.
H. J. Warmuth, Smyrna, Tenn.
L. W. Fox, Philadelphia, Penn.
James B. Murfree, Murfreesboro, Tenn.
Compbell Slayden, Pinewood, Tenn.
John S. Mabon, Alleghany City, Penn.
Cochran McClelland, Philadelphia, Pa.
H. O. McConnel, New Brighton, Pa.
Wm. H. Pancoast, Philadelphia, Penn.
Joseph S. Miller, York, Pa.
S. M. Ross, Altoona, Pa.
Thos. L. White, McKeesport, Pa.
John V. Shoemaker, Philadelphia, Pa.
E. O. Shakespeare, Philadelphia, Pa.
Alvin Thayer, Erie, Pa.
George E. Stubbs, Philadelphia, Pa.
John Sandt, Castor, Pa.

Theo. P. Simpson, Beaver Falls, Pa.
Jefferson H. Wilson, Beaver, Pa.
W. S. Stewart, Philadelphia, Pa.
Thos. A. Shaw, Pittsburgh, Pa.
Wm. C. Wile, Philadelphia, Pa.
Al. A. Seem, Bangor, Pa.
A. K. Seem, Martin's Creek, Pa.
Frances M. Perkins, Philadelphia, Pa.
Chas. S. Musser, Aaronsburg, Pa.
H. W. Hitzrat, McKeesport, Pa.
Wm. Dickey Kearns, Pittsburgh, Pa.
Jacob S. Hackney, Uniontown, Penn.
J. M. Gemmill, Jr., Tyrone, Pa.
M. O. Jones, Pittsburgh, Pa.
J. W. Holland, Philadelphia, Pa.
R. P. Heilman, Emporium, Pa.
Alexander M. Pollock, Pittsburgh, Pa.
J. C. Dunn, Pittsburgh, Pa.
E. Allen Wood, Pittsburgh, Pa.
Charles W. Dalles, Philadelphia, Pa.
W. T. Bishop, Harrisburg, Pa.
Wm. B. Atkinson, Philadelphia, Pa.
Wm. M. Brinton, Sharpesburg, Pa.
W. H. Daly, Pittsburgh, Pa.
Wm. Anderson, Indiana, Pa.
Wm. S. Foster, Pittsburgh, Pa.
J. B. Caldwell, New Hamburg, Pa.
Stephen A. Craig, Freedom, Pa.
G. Y. Boal, Baden, Pa.
W. Earnest Good, Philadelphia, Pa.
E. S. Gans, Philadelphia, Pa.
Jno. Wiest, York, Penn.

RHODE ISLAND.

Wm. J. Burge, Pawtucket, R. I.
E. S. F. Arnold, Newport, R. I.

SOUTH CAROLINA.

Thos. Legare, Charleston, S. C.

TENNESSEE.

W. C. Cook, Nashville, Tenn.
Chas. N. Cooper, Cleveland, Tenn.
Wm. Y. Ewing, Nashville, Tenn.
Wm. F. Briggs, Nashville, Tenn.
John H. Callender, Nashville, Tenn.
Giles C. Savage, Nashville, Tenn.
J. B. W. Nowlin, Nashville, Tenn.
Jas. B. Stevens, Nashville, Tenn.
Ed. Duncan, Nashville, Tenn.
Wm. F. Glenn, Nashville, Tenn.
S. T. Armstrong, Memphis, Tenn.
D. J. Roberts, Nashville, Tenn.
Richard Cheatham, Nashville, Tenn.

TEXAS.

W. P. Burts, Ft. Worth, Tex.
O. Eastland, Wichita Falls, Tex.
R. W. Park, Waco, Tex.
M. A. Taylor, Austin, Tex.
Daniel B. Blake, Cusco, Tex.
Wm. A. Adams, Fort Worth, Tex.
Charles H. Sears, Waco, Tex.

UNITED STATES NAVY.

Delevan Bloodgood, U. S. Navy.

VERMONT.

Samuel H. Griswold, Rutland, Vt.
M. R. Crain, Rutland, Vt.

VIRGINIA.

Lawrence Ashton, Falmouth, Va.
J. E. Chancellor, University of Virginia, Va.
Herbert M. Nash, Norfolk, Va.

WEST VIRGINIA.

J. E. Reeves, Wheeling, W. Va.
Chas. M. Frissell, Wheeling, W. Va.

WISCONSIN.

George V. Mears, Fond du Lac, Wis.
Jno. R. McDill, Milwaukee, Wis.
Lucy Eastman Ermine, Royalton, Wis.
John S. Walbridge, Berlin, Wis.
George Leiler, Alma, Wis.
Arthur J. Bergess, Oshkosh, Wis.
Samuel Bell, Beloit, Wis.
F. T. Nye, Beloit, Wis.
Eugene H. Townsend, New Lisbon, Wis.
G. M. Steele, Oshkosh, Wis.
J. Ashley Thompson, Oshkosh, Wis.
Frank S. Wiley, Fond du Lac, Wis.
G. F. Wilter, Grand Rapids, Wis.
Henry Palmer, Janesville, Wis.
Daniel Seymour McArthur, La Crosse, Wis.
Edward Martin McDonald, Doylestown, Wis.
David W. Moore, Waupun, Wis.
Darwin L. Manchester, Waupaca, Wis.
Wm. T. Pinkerton, Eau Claire, Wis.
B. O. Reynolds, Lake Geneva, Wis.
Hugo Phieler, Waukesha, Wis.
Samuel William French, Milwaukee, Wis.
John N. O'Brien, Milwaukee, Wis.
Geo. T. Dawley, Royalton, Wis.
David C. Davies, Columbus, Wis.
Robert W. Earl, Columbus, Wis.
John G. Meachem, Racine, Wis.
T. P. Russell, Oshkosh, Wis.
F. R. Garlock, Racine, Wis.
L. H. Hayman, Boscobel, Wis.
Christian Linde, Oshkosh, Wis.
Albert Hayden, Shullsburg, Wis.
J. C. Hayward, Marshfield, Wis.
J. K. Bartlett, Milwaukee, Wis.
N. Monroe Dodson, Berlin, Wis.
W. Eastman, Mineral Point, Wis.
Wm. T. Galloway, Eau Claire, Wis.
R. H. Bingham, Oshkosh, Wis.
I. W. DeVoe, Wausau, Wis.
Henry L. Day, Eau Claire, Wis.
B. C. Guddan, Oshkosh, Wis.
Wm. L. Tallman, Mineral Point, Wis.
David La Count, Chilton, Wis.
Chas. Alexander, Eau Claire, Wis.
J. F. Corbett, Weyanwega, Wis.
A. H. Guernsey, Amherst, Wis.
J. Cavaney, Milwaukee, Wis.
Edward H. Irwin, Lodi, Wis.
John Phillip, Stern's Point, Wis.
J. W. Hawes, Milwaukee, Wis.
R. M. Hawley, Milwaukee, Wis.
Geo. E. Catlin, Lake Geneva, Wis.
Geo. R. Vincent, Wis.
Geo. Jenkins, Kilbourn City, Wis.
John B. Edwards, Mauston, Wis.

WASHINGTON TERRITORY.

N. F. Essig, Spokane Falls, W. T.

### FIRST DAY'S SESSION.

Prayer was offered by Dr. McPherson, pastor of the Second Presbyterian Church of Chicago.

Dr. Smith, in introducing Mayor Roche, said it is many years since the Pottowottamie Indians had encamped on the banks of the Illinois river. Ever since that time a strange medicinal odor had embraced the stream; and, in the evolution of time, the American Medical Association meets in Chicago. We bid you welcome to our city, and, in extending that greeting, I have the pleasure of introducing to you Mayor Roche.

### THE MAYOR'S ADDRESS.

Mayor Roche responded as follows:

Mr. President and Gentlemen, Representatives of the Science of Health and Life: In the name of the citizens of Chicago, I welcome you to this city, distinguished for the large number of able and eminent members of the medical profession, and for the exemplication in all the evocations and pursuits of life of the precept, "Whatsoever thy hand findeth to do, do it with thy might." Your mission—to preserve health and remove disease, to prolong life and make it a blessing—is a beneficent and noble one, worthy of all honor. And, though you have not yet succeeded in overcoming death, you have robbed it of half its terrors.

The present generation has seen great progress in medical science; and the medical profession, I think, has kept pace with the other learned professions, if it has not even excelled them, in original investigations and practical discoveries for the benefit of mankind. (Applause).

When in health we laugh at the doctors, and sometimes enjoy a joke at your expense. But in sickness you are our hope and refuge; and, to the worn and wasted patient, just struggling back to life from the gates of death, you are like "the shadow of a great rock in a weary land."

The interchange of ideas and experience, and the discussion of theories and experiments by large bodies of educated men, gathered from different and distant sections of the country, by which the individual thoughts and knowledge of each become the common property of all, is a comparatively modern outgrowth of society and must contribute greatly to the interest and usefulness of the medical profession, being full of promise for the future. (Applause.)

These gatherings for mutual comparison and consultation minimize differences, soften asperities, cultivate the amenities, strengthen the humanities, stimulate inquiry and investigation, extend the horizon of mental and moral vision, enlarge the boundaries of human knowledge, and tend to the unification, improvement and well-being of the whole community.

Gentlemen: I came here as the official representative of a great and hospitable city, whose latch-string is always out, to emphasize the welcome of Chicago to this large, intelligent and representative convocation of a profession whose chief occupation is to *save* life and not *destroy* it, and whose cardinal doctrine is that a sound mind in a sound body is essential to the best performance of the duties of this life, and a great help in fitting men for the life hereafter.

The Chairman of the Committee of Arrangements gave notice that several papers had arrived too late to appear on the programme but would be referred to the appropriate sections.

One by Dr. DeLaskie, of Chicago, on "Infant Feeding" another by Geo. W. Jones, of Danville, Ill., on "Diarrhea in Children," a paper for the surgical section by Dr. Dulles, of Philadelphia, and "The Report of the Committee on Medical and Sanitary on Service on Board Immigrant Passenger Vessels" by Dr. A. N. Bell, of New York.

### PRESIDENT'S ADDRESS.

The Chairman then introduced the President, Dr. E. H. Gregory, of St. Louis, who addressed the Association as follows:

Obviously, force and matter "make up" the universe. Force implies antagonism, antagonism perpetuates motion. The living cell is the embodiment of nature. Cell antagonism is life. Multicellular organisms represent a community of unities. A model organism in equillbrium is health. Cell struggle is the gist of modern pathology. Every organ and every element are vulnerable. The strength of resistance in elements and organs, reinforced by the harmony and precision of co-ordination and the vigor of counter agencies, are the momentuous questions.

In seeking for a comprehensive title for my address, I fell upon the two simple words "Cell Antagonism," which form the foundation of my symptomatology and pathology, conjoined with cell changes, the basis of pathological anatomy, embracing at once the universe of life and all the possibilities of life; disease being but one of multitudinous phases of life.

Huxley has likened the body to an army. "Of this army, each cell is a soldier; an organ, a brigade; the central nervous system, headquarters and field telegraph; the alimentary and circulatory system, the commissariat. Losses are made good by recruits born in the camp, and the life of the individual is a campaign, conducted successfully for a number of years, but with certain defeat in the long run." A model organism assumes that every soldier is a stalwart; every organ a body of stalwarts, with corresponding machines of co-ordination and precision, all alike concerted and vigorous to meet the myriads of counter forces, many of which are too cunning for our ken, but ever ready to take advantage of the least failure of our strength to penetrate our vitals. This force of vital resistance forms the basis of therapeutics, this "vis mediatrix naturæ" will be always the indispensable auxiliary of the physician, without which ally abandonment of the art would be inevitable.

The morophologist distinguishes two classes of elements within the organism. One class represents the fixed cells, cells which occupy the fortifications within the inter-cellular substance; the other class provides for the formation of new tissue, and represents the standing army, sometimes called embryonal substance. Before Cohnheim, our knowledge of the mobile elements—the mobilized army—of the body, was nothing; now most of the literature relating to neoplasm is or has become obsolete. Now coagulable lymph is impossible without the white corpuscles. Organizable fluids have vanished. Through the ingenuity and industry of the great German pathologist a revolution has been worked in medical thought. The study of the mobile elements of the body—the relatively independent elements—promises much. We can almost see daybreak in pathology, a dawn before the coming light which is to illuminate some of the dark places of this most intricate subject. Already leucocytes substitute fibrin. The latter is the product of the former. Wherever there is localized impairment of nutrition, the result of inherent weakness, the leucocytes concentrate, feast on the devitalized structures, and thereby develop into a tissue replacing that which they have consumed. When the surgeon wishes to dispose of an adventitious structure—a cyst, for example—in the connective tissue, he lowers the vitality by over-stimulation—irritation—when he confidently expects the leucocytes to congregate and destroy the cyst, substituting a granulation tissue, which in time becomes scar tissue. Now an organizable fluid as the essential product of inflammation, cells playing a passive part, is a thing of the past. Now cell potency, cell subtlety and cell antagonism constitute the pith and marrow of medical science. There was a period in the history of pathology when all the neoplasia sprang from the tissue cells; now the place and destiny of the tissue cell is fixed. When the tissue elements yet contain some undifferentiated protoplasm, proliferation is possible, otherwise not probable. The mobile cells constititute the mobilized army; the great co-ordinating centers assemble them at any moment, ready to antagonize hurtful agencies. Irritation always provokes a concentration of the white cells. Irrigation means injury. It may be simple or specific. Either way it is a signal for moving the army in solid phalanx to confront noxious agencies, and the combat is hotly contested till victory or discomfiture ensues.

Inflammation, the keystone of medical science, the standard process by which all other pathological conditions are measured, is only to be explained on the theory of cell antagonism; it is practically a struggle between irritant bodies and white blood corpuscles. Foreign bodies are invariably invaded by leucocytes, their swarming having for its object to repel or render inert the offending cause. Observations directed to the white cell have been followed by an inspiring increase in our knowledge of the vital properties of cells in general. The quality of spontaneous motion once accepted, the idea that the cell played a passive part quickly disappeared, and in its stead came the conception of autonomy, the fact that the cell specialized food for its own purposes, actually breaking up compounds and adapting the products to its own growth and development, strikingly illustrated by an independent form of government—for example, the government of the United States. A great statesman has said ours is a "government of the people, by the people and for the people." Cells live of themselves, by themselves and for the whole body. The cells make the central government. The coördinate machinery is formed by the cells, and the cells are in their turn centralized and harmonized by this power. The tissue elements are in direct continuity with the centre; but it is otherwise with the floating, wandering cells; yet their behavior gives evidence of their concern for the well-being of the entire organism. Again, the free cells take into their substance the most refractory substances, digesting solid bodies; sponges and green plants yielding to their digestive and assimilative processes. Observations the most patient and trustworthy in the lower vegetables have demonstrated the fact that bacteria have been seen in the substance of amœboid cells; others have been seen pursuing bacteria, ultimately capturing and destroying them. Throughout the whole animal kingdom mesoderm cells use their ingestive power for destroying micro-organisms. Further, this property seems to be utilized for the removal of larv organs. Colorless corpuscles present the same appearance and have similar properties and the same mode of origin in the entire range of living beings. When the irritant or harmful agency is particularly obdurate, white cells exhibit a very strange habit of throwing out processes which unite with similar processes from neighborlng cells, until a considerable mass of protoplasm is formed by their confluence, constituting a giant cell. Thus an army of giants may be improvised on occasions of extreme emergency; for example, when the tail and gills of the tadpole are to be swept off, this powerful division is ready for the Herculean task.

Apply these facts to the inflammatory process in mammais. Suppose a foreign body lodged; that moment leucocytes leave the blood vessels and congregate for the purpose of opposing the intruder. In such diseases as tubercle, leprosy, etc., the giant cells appear, and in their center—their substance—the specific bacillus is found. Koch found the bacillus of anthrasis and the bacillus of septacæmia in the mouse, enclosed by white blood calls. We say, with these obvations before us, inflammation exhibits a new aspect; cells conquering cells as a process of normal physiology; simply the free cells, the white cells resisting injury, there being a provision not only for concentration, but for increasing their number and augmenting their strength, corresponding to the emergency. A recent authority, Mr. Sutton, says: "If we summarize the story of inflammation as we read it zoologically, it should be likened to a battle.. The leucocytes are the defending army; their roads and lines of communication the blood-vessels. Every composite organism maintains a certain proportion of leucocytes as representing its standing army. When the body is invaded by bacilli, bacteria, micrococci, chemical or other irritants, information of the agression is telegraphed by means of the vaso-motor nerves, and the leucocytes rush to the attack, reinforcements and recruits are quickly found to increase the standing army, sometimes twenty, thirty or forty times the normal standard. In the conflict cells die and often are eaten by their companions; frequently the slaughter is so great the tissues become burdened by the dead bodies of soldiers in the form of pus, the activity of the cells being testified by the fact the protoplasm often contains bacilli, etc., in various stages of destruction. The dead cells, like the corpses of soldiers that fall in battle, later become hurtful to the organism they in their lifetime were anxious to protect from harm, for they are fertile sources of septicæmia and pyæmia, the pestilence and scourge so much dreaded by operative surgeons. The analogy may seem to some a little romantic, but it appears to be warranted by the facts.

Just here the question obtrudes, Is the action which follows mechanical, chemical and thermal injuries, and the action caused by vital injuries alike inflammatory processes? In the one, that resulting from physical causes, the nutrient processes are not disturbed, simply increased. In the other, that resulting from vital causes, the cell processes are disturbed, thwarted and vitiated. It seems unfortunate that the action which succeeds to traumatism should be confounded with that which succeeds to specific germs. The nutrient changes come into vigorous play after an ordinary injury, altered in one particular only, viz: the presence of a palpable neoplasm, which substitutes the structures doomed by the injury. The addition of new material is invisible in ordinary nutrition, growth and maintenance for example, the substitution being atomic. When a structure is destroyed bodily, as in traumatism, the substitution is corpuscular, therefor palpable. It is certainly perplexing to assume, as does Sir James Paget, that a visible neoplasm is produced by inflammation, but its development is impossible till after the withdrawal of the inflammation. To get rid of confusion, though we do not emerge from error, is to make a step toward truth. The full effect of traumatism is immedtate and complete; it cannot increase itself, therefor its effects are always limited and within the possibility of estimation, for the reason that cause and effects are in precise correspondence; besides antiseptics do not influence the changes in any particular. On the other hand, the most extensive and severe injuries of vascular parts, involving bone, joints, the great cavities, etc., is possible without producing inflammation, provided antiseptic precautions be taken. When such complication ensues, it is a wound accident, not a wound incident, and the result of infection. There is no disturbing factor in the process incident to repair; no waste products. If pus is found, it is the result of the intrusion of some noxious agency, some contagion, some pestilence; in short, a specific cause.

If it be agreed to call the action inseparable from ordinary injury physiological repair, the term inflammation is limited to the series of events referable to a vital agency, which until very recently has eluded detection. A specific agency determines a class of diseases as distinctive and definite as the living creatures studied in natural history. Cell antagonism after physfcal injury is simply a substitution, through the development of mobile cells, of the tissue which has been spoiled. The fixed elements multiply and develop in the expansion and perfection of the several structures of which they are components, but take no active part in the process of repair after injury. After the perfection of the body, the factors favoring proliferation and those which inhabit it must be in a state of balance. In functional hypertrophy, the balance is toward the side of proliferation. On the other hand, in non-functional hypertrophy, the problem is not so simple; the balance is disturbed toward proliferation by some mysterious agency. How strikingly different when the cell antagonism is with any vital injury; here cell mysteries clash. The bacteriæ enter into conflict with mobile and fixed cells, but it is not possible to know how the conflict is carried on. Certainly a series of disturbances ensue in the normal metabolism of the elements. The functional, formative and nutritive activities, which are the expressions of cell life, must be altered; their vigor, perhaps, lessened, their susceptibilities modified. Exceptionally their life is enfeebled or extinguished. The issue of a bacterial affection is either the death of the patient or the destruction and elimination of the bacteriæ. It follows that this disturbance of cellular activities is always at the bottom of morbid symptomatology; and observations have shown that disease of this kind, successfully withstood, leaves the element in a peculiar unsusceptible condition, insuring an immunity, almost or quite complete, against a fresh invasion of the same or kindred bacteriæ. This modified susceptibility was practically understood by Jenner. Pasteur, resuming and systematizing the great Englishman's work, successfully modified cell forces, rendering them harmless by cultivation, and sending them on the important mission of destroying the natural proneness to the deadly assaults of the uncultured cells. Whilst we may despair of ever understanding the essential nature of vitality, the study of the causes which regulate life, and their subordination to conditions which may be determined, has led the way to the grandest achievements of recent times. Pasteur and Lister are the great apostles of modern practical thought.

Huxley has suggested that it may be possible to introduce into the economy a molecular mechanism which, like a cunningly contrived torpedo, shall find its way to some particular group of cells and cause an explosion among them, leaving the rest untouched. Pasteur's cell mechanisms exceed the conception of the great scientist, for they cunningly change the vital qualities of the elements without desroying them. May we not look forward for some great advance in therapeutics in the direction to which Pasteur's geuius points, by the study of cell antagonism? Certainly vital subtleties may best cope with vital subtleties. Study of the conditions in which infective agents arise, by ascertaining the circumstances which limit or facilitate their diffusion, has already raised surgery to a proud preeminence. With therapeutics it is otherwise, as it is doubtful whether the new facts have yielded the slightest increase of power against the diseases of mankind. Let us not despair. Knowledge must come first, then wisdom brings its practical application. The study of cell possibilities, their readiness and energy in rendering inert noxious agencies, whether introduced from without or arising from within, exhibiting an antagonism at once potent and direct, rather tends to dampen one's therapeutic enthusism. The thoughtful student sees nothing abnormal in disease. To be sure there is a physiological emergency, but there is no disorder in the cell processes; rather the perfection of order. If a physical or chemical cause intensifies the cell changes, in other words, determines the physiological emergency—every cell movement is direct, purposive and efficient, ending only when the intruder is ejected, encysted or accommodated. On the other hand, if the offense be a vital one, a living cell, a microphite, the spectacle is that of one living creature preying upon another, the declaration of the first law of nature, not an enemy, but an intruder struggling for self preservation, simply a physiological fight for life. Can we hope an ideal tonic for cell antagonism, such as would innervate the cells on one side, and enervate them on the other, the "old chestnut," "Prevention is better than cure," is not yet worm eaten.

Cell antagonism implies a struggle. The duration of the struggle is determined by the quality of the irritant and the strength and resources of the antagonists. Acute inflammation is a sharp and decisive action, chronic inflammation is slow and indefinite. There are two striking, important chronic conditions, viz: The interstitial inflammation and the infective granulomata, interesting alike to the physician and the surgeon; both are alike disastrous, the one, interstitial inflammation, distroying by cicatrical contraction; the other, infective granulomato, by stopping at the fibro-blast stage, retrogression replacing development. The quality of infection and the failure to develop beyond the stage of granulative tissue is the exact condition, as illustrated by tuberculosis, syphilis, lupus, etc., in contrast with which is fibrosis of the liver, cirrhosis of the kidney, Bright's disease, fibrosis of the brain, sclerosis, interstitial inflammation, destroying the tissue clements by strangulation and infective granulomata by contamination.

Again, the two varieties of inflammation referred to declare that the quality of the irritant determines the effect of the inflamation, the interstitial variety being caused by physical agency, and the infective by a vital one. Cells antagonize physical causes without losing any of their vital qualities. Not so when cells antagonize vital causes. When cells are pitted against cells they may be despoiled of their highest quality, as in the infection granulomata they have parted with formative activity. In short, the knowledge that pertains to the presence of a vital irritant epitomizes all that has been taught of infective disorders. The symptomatology and pathology of this class of diseases is but the life history, the play of cell activities, metabolisms and catalysis of antagonizing organisms, as likewise to the knowledge pertaining to the presence of a foreign body or physical irritant is the knowledge, in brief, of fibrinoses in general of that entire class of disorders known as interstitional inflammations. A persistent irritant—injury—with its attendant concentration of leucocytes, their inevitable development and ultimate transformation, the history of the capsulation of a foreign body, whether it be animate or inanimate is the history of cirrhosis of the liber, fibrosis of the lung, morbus Brightii, atrophy of the heart, atrophy of voluntary muscles in general, sclerosis of the brain and bones; in short, diffuse capsulation. Some disseminated irritant, say alcohol, determines a sclerosis of the liver, and how? Every particle of alcohol being a foreign particle, having its capsule, it follows that capsulation is as uniformly distributed as the cause. An artisan inhales fine particles of steel or other foreign material, and fibrinosis of the lung is the consequence. The irritants of gout and rheumatism are lodged in the kidney, the interstitial tissue of the organ increases, and in the end strangulates the tubules and malpighian bodies, resulting in shrinking and total disorganization of the organ, constituting Bright's disease. Like changes occur in the heart co-incident with the development of the interstitial fibrous tissue, as atrophy of the muscular substance, substituting a fibrous induration for its normal structure. A similar change in the voluntary muscles occurs in the curious disease known as the pseudo-hypertrophic paralysis, chiefly afflicting children. The connective tissue between the muscular fibres increases so much that the muscles affected may exceed their normal size three times. Later, however, the new tissue shrinks, and the contractile material of the muscles is spoiled. The condition of sclerosis in bones corresponds to cirrhosis of the liver, and Bright's disease of the kidney. In the nerve centers of the interstitial tissue, neuralgia takes on the same chronic over-growth, strangling the nerve strands and cells, giving rise to the most singular and complicated nervous phenomena. The changes thus induced are recognized by the general term sclerosis. If it involves the fascicules of Burdack and the column of Goll, locomotor ataxia results, if the medulla, bulboo paralysis, etc.

We have purposely avoided reference to diathesis, as also to the precise neurological relationship of cells, not because we deny their influence, but because too little is definitely known. On the contrary, the behavior of cells relatively to irritants is well understood. The presence of a foreign body is at once and directly resisted by the leucocytes, a zone of inflammation, its destruction, transportation or capsulation, even parasites which resist the death-dealing assaults of the leucocytes are at last imprisoned in cases of fibrous tissue.

There yet remains for our consideration a mysterious possibility relating to cell life of great practical moment, viz.: that of entering upon a life of independence, separating from the central nervous system "headquarters," disregarding the "field telegraph," oblivious to the morning "drumbeat," and wholly ignoring the restraining and directing influence of environing structures. We have learned that however much irritation may affect the vital qualities of cells, their fidelity is always preserved, there being no sign of disaffection, the army ever intact, the fealty of the soldiers supreme. Physical causes—traumatism—may crush or impair the tissues, but the cells come to the rescue with all vigor and directness. The prolonged presence of bacteriæ may impair the forces of the cells, enfeeble and extinguish them, but as long as life lasts their efforts are in harmony with the purposes of the organism. Thus it appears that alienation is not possible through the agency of irritation, however intensified, modified or prolonged.

The question constantly obtrudes: Is alienation possible to a mature element, to an element that has assimilated itself physiologically as well as anatomically, with the surrounding tissues; taking part in its functions; concurring and cooperating in all the processes of the economy? This question seems to have confronted Cohnheim when his ingenuity suggested the embryonal hypothesis, viz: that the tumor germ was congenital; that there were in the mature body embryonal elements and tissues not utilized in the elaboration of the normal structures; latent, embryonal rudiments; small embryonal cells so diminutive as to elude observation, inactive perhaps, till some exciting cause awakens activity. Is not an embryonic element in a mature organism an alien? Is it not already an independent element? Are not the patches in the skin, pigmented moles, islands of cartilage in mature bone, congenital angloma, etc., independent structures, already tumor germs, congenital rudiments of tumors? Accept the hypothesis of the great German pathologist, and the problem of tumor etiology is almost solved; it remains only to find the exciting cause; the predisposition is inherited; irritation, simple or otherwise, may be nearly allied to the awakening impulse. The difficulty is to reconcile cell antagonism with the proposition this doctrine includes; the idea that all discordant bodies are treated as intruders, either ingested, transported or covered up. We can conceive the possibility of the existence of an inoffensive tissue or element, one that simply draws its nourishment from adjacent tissue, without any serious disturbance of their normal metabolism. We would gladly adopt the theory of Cohnheim, which ascribes the origin of tumor neoplasms to persistent germinal rudiment Certainly it has much to recommend it. Countless latent embryonal structures, relatively independent, are tolerated by the organism, remaining inactive till favored by some concurrent event, the nature of which is incomprehensible, when they may grow according to their bent. Is it not quite as possible there may be similar, relatively independent elements, ready when stimulated, to multiply independently? Congenital angloma often grow without definite limit; pigmented moles; black, slightly-raised patches in the skin, with which all are familiar, are composed of tissue exactly resembling sarcoma, always suspicious because of their disposition to proliferate independently. It is most difficult to believe in the infidelity of covenant cells, of sundering the compact which unifies the organism. Emancipation seems almost out of the question. We must not only believe that dependent benignant elements become the elements of a parasite—a parasite so vigorous, corrupt and wicked as to destroy its source.

The painful part of the retrospect of a year is that which recalls those who have been taken from us. The past year has had a very special grief for our association in the death of one of our ex-Presidents, Dr. William Owen Baldwin, who died at his residence, and the place of his birth, Montgomery, Alabama, May 30, 1886, He was among the early members of the American Medical Association, and its President in 1869, residing at New Orleans. His address on that occasion will be remembered as abounding in Christian and patriotic sentiments, eminently befitting the good and great physician. The Committee charged with the organization of the Ninth International Medical Congress, which meets at Washington City this year had named him as one of its Vice-Presidents. Dr. Baldwin was remarkable for his culture, the eloquence of his speech and the beauty of his diction, and will be remembered as an imposing figure in the history of this great Association.

I need scarcely remind you, gentlemen, that we shall have with us, after a few weeks, the medical men of all nations. Soon we shall extend the hand of fellowship to those with whom we have heretofore been united in interest and sympathy in the cause of science. We know that everything is made ready and that success is assured. Again, you will join me, I know, in the declaration that a hearty American welcome awaits their advent, and that the Ninth Meeting of the Great Congress will be memorable in the history of its organization.

After considerable discussion it was decided that the following report should be made to the Association as whole: The Report of the Committee on Medical and Sanitary Service on Board Immigrant Passenger Vessels." This report would have been submitted last year but for a delay in mail delivery.

After considerable correspondence with the House Committee on Commerce the Committee found that the "Act to Regulate the Carriage of Passengers by Sea" of July 22, 1882, was supposed to provide against the evils complained of. But the Committee felt certain amendments to be necessary, and addressed a letter January 21, 1886, to Hon. John H. Reagan, Chairman of the House Committee on Commerce, calling his attention to the ways in which the obligations of the law are evaded.

The law reads "whereon emigrant passengers, or passengers other than cabin passengers," and first cabin and saloon passengers are construed as being exempt from legal obligation, and the Committee also calls attention to the incompetency and disgraceful status of the medical officers and recommends these amendments to meet these evasions.

1st. Wherever the words "cabin passengers" occur, they should be made to read first cabin and saloon passengers.

2nd. In section No. 5 requiring that "Every steamship or other vessel carrying emigrant passenger, or passengers other than cabin passengers exceeding fifty in member, shall carry a duly qualified and competent surgeon or medical practitioner," after "practitioner" to add that if the number of such passengers and crew is over six hundred, a junior or assistant surgeon or medical practitioner be added; the service of such practitioners to be given promptly, without fee, in every case of sickness, and whether there be one or two they be required to perform the duties of sanitary officer, reporting to the Master of the vessel with all suggestions needful.

They should be provided with a competent apothecary or steward, and it shall be their duty to report in writing, under oath, to the officer of the port at which they arrive a detailed account of all sickness and every case of insanity or imbecility noticed. The Committee did not feel called upon to touch on the failure of that portion of the bill intended to provide against the immigration of convicts, etc.

The Session then adjourned.

---

THE SECTION ON SURGERY AND ANATOMY

was very largely attended. In the absence of the Chairman, the Secretary, Dr. Pollock, presided, Dr. Walker acting as Secretary pro tem.

The paper of Dr. Summers on Gerebral Abscess' was passed and referred to the committee for publication. Dr. S. C. Armstrong read this paper on "Trephining in a Case of Intermeningeal Hæmatoma with Hemiplegia."

Dr. Walker, in discussing the paper, reported the case of a man brought to the hospital by the police, who supposed him to be suffering from intoxication. His left arm and leg were paralyzed. After remaining in this condition for five days, a conical incision was made over the right fissure of Kolando, and two buttons of bone and four ounces of coadgulated blood were removed. A rapid recovery followed, and he left the hospital cured at the end of three weeks.

"SCIRRHUS OF THE MAMMÆ."

Dr. W. C. Wile, of Philadelphia, read an exhaustive paper on the above subject. His treatise is so excellent that we give an almost literal abstract of it. He said:

Which of you have never been approached by a timid, shrinking woman, who, bearing her breast as though making ready for the sacrificial altar, stands awaiting her doom which shall hang upon your verdict? Which of you, think you, know of the agony this poor being has already passed through? Who of you can guess the anguish and fear that have finally driven her to seek a confirmation for her one absorbing thought—the phantom that stays by her and is her inseparable companion by day and by night? Have you ever looked into her eyes when she asks, "Doctor, is it a cancer?" They tell us the eye is the mirror of the soul. Oh! what unutterable depths of anguish do they not depict, when finally, after an examination, we are reluctantly forced to admit the confirmation of her worst fears. How they shine with gratitude, thanksgiving, and joy if we may positively assure her the grounds are unfounded. In my whole medical career I have never found a disease that holds such a frightfully depressing sway over a patient's thoughts—one that is so infested with terrors for them, that, apart from all pain, has the power of so ruining a bright, happy, lovable, womanly nature as scirrhus of the mammæ. I have known a woman with cancer of the breast go about her daily vocation, attending to duties, social and wifely, that her position demanded, suffering the agony of the damned, and refusing all treatment, rather than give to her husband and children the knowledge that she suffered from a mammary tumor. Every atom, then, that we may bring to bear testimony to a substantiation of older modes of treatment, any statement we can truly make to fortify good results, should be freely given, so that it be in our power to offer these poor sufferers the effects and benefits of what interference we shall practice.

I have nothing new to offer you in my paper; but I wish to reiterate a point or two that previous writers have already offered, and which, in my own experience, now stand strongly approved. It is not my purpose to enter into the etiology, pathology, or diagnosis of scirrhus, but simply content myself by saying the first is always doubtful, the second well known, and the latter never sure until a section is laid under the microscope. Believing this, then, so firmly, I may say, in the twenty-five cases I present to you, I have had sections of the removed tumors made and a diagnosis of their character given by some able microscopist. In justice to myself and many others I know, I may say, I have removed tumors for scirrhus cancer that subsequent investigations have not verified, and again tumors that were diagnosticated as benign have been found to be malignant growths. This is, of course, not of frequent occurrence; but the fact that it does happen, and to the best of us, is what led me to say, a moment ago, that there is always a doubt as to the absolute truth of the diagnosis until verified by microscopical investigation.

With respect to treatment there is now, as there was many centuries ago, diversity of opinion as to what interference shall be practised; in my own humble opinion and that of the vast majority of surgeons there is but one mode of procedure. It consists of amputation, and the earlier the amputation the more likely are the beneficial effects to be enhanced. The fact that a scirrhous cancer will at very rare intervals remain in an adolescent state is no proof or, rather, evidence against the justice of early operative interference. When the patient is advised to wait for developments, we may justly say it is done to cover the ignorance of the attendant, or evidently the result of a timidity which he cannot overcome, and results in an amount of unnecessary distress which might well be avoided. This every unbiased practitioner will verify, for our results cannot be measured by single instances or by the reports of a few fortunate operators, but must be massed and the inferences drawn from the aggregate cases. Prof. S. W. Gross found the average duration of life in cancer of the breast, if allowed to pursue its own course, to be twenty-seven months. Where extirpation was practised the average was raised to thirty-nine months, a clear gain of one year. With regard to an ultimate result an analysis of 542 cases by the same observer showed that fifty-seven survived over three years without any recurrence, and died of an intercurrent malady, and fifty-three were alive at the date of last reports. The average duration of life after the operation was six years and five months, while only 1.5 per cent. of the cases allowed to run a natural course survived six years. Of the cures 31.57 per cent. were free from the disease at the end of six years. The average duration of the disease in these cases had been seventeen and a half months—as you can see, a period too late to be in its most favorable aspect.

The age at which carcinoma of the mammæ most commonly occurs is about between the thirty-fifth and fiftieth years of age. The youngest cases reported of which I have any knowledge are those of Mr. Lyford and Mr. B. B. Cooper. Lyford's case was a girl of eight years; Mr. Cooper's was thirteen years old. Holmes speaks of a preparation, in St. Bartholomew's Hospital, which was removed from a maiden of sixteen. Prof. S. W. Gross' youngest case was twenty-seven years old. Holmes reports 458 cases in which the disease occurred at the following ages:

| AGE. | | AGE. | |
|---|---|---|---|
| From 20 to 30 | 19 | From 60 to 70 | 34 |
| " 30 to 40 | 100 | " 70 to 80 | 6 |
| " 40 to 50 | 193 | " 80 to 90 | 7 |
| " 60 to 60 | 97 | " 90 to 100 | 2 |

Thomas Bryant reports the ages of 400 cases as follows:

| AGE. | | AGE. | |
|---|---|---|---|
| Under 30 years | 17 | From 50 to 60 | 101 |
| From 30 to 40 | 108 | " 60 to 70 | 29 |
| " 40 to 50 | 144 | Over 70 years | 1 |

Of the twenty-five cases which I report to-day the age was as follows:

| AGE. | | AGE. | |
|---|---|---|---|
| Under 30 years, | 1 | Between 50 and 60 | 5 |
| Between 30 and 40 | 6 | " 60 and 70 | 1 |
| " 40 and 50 | 11 | Over 0 years | 1 7 |

Death as a result of the operation is rare, and occurs only from shock when the debilitation of the disease has assumed grave proportions. In the case of death which I report the operation was performed against my better judgment, for I allowed my refusal to be overruled by the persuasions and entreaties of the patient and her friends. I think, as a general thing, it is a fair statement to say: never operate on a patient who has passed her seventieth year, and never operate when atheromatous degeneration exists; they always do badly. Just one century ago Benjamin Bell wrote: ". . . . Even in very advanced stages of cancer it is right to amputate, provided the parts affected can be completely removed. When, indeed, this cannot be effected, from the cancerous parts lying too deep, or from their being immediately connected with organs essentially necessary to life, by which amputation of the one cannot be performed without considerable injury being done to the other; in such circumstancss—as the operation would not be of any real utility—it should not be recommended: for, as all the diseased parts could not with propriety be removed, and as the cancerous virus is of a very assimilating nature, it would answer no beneficial purpose to amputate only a portion of them. But in every instance when the parts affected can be safely separated from the sound, as nothing but their removal can afford any chance of safety, we must again say that no hesitation should occur in advising the operation." So wrote this great surgeon a century ago, and to-day I have, after all my studies, with our greatly increased facilities for pathological research, and the improvements in operative procedure, nothing to add, nothing to detract from the great truth he has embodied in the foregoing words.

Another point to which I wish to strongly draw your attention is the unnecessary slaughter to which the skin is subjected in these amputations. Where the skin is not involved save it, and a grand rule is, save all of this tissue that can possibly be saved. The less skin removed, the more readily does the wound heal. The smaller the cicatrix, the less likelihood for a residive in the original site. I find Bell remarked this, and speaks of it in the following terms: "But when any portion of the skin is taken away, . . . . the remaining cicatrix is necessarily of the same size; a tenderness is left in the site of the disease, which I am convinced has often some influence in giving rise to a return of it." Later on, he says he never had cause to rue this saving, which he practiced for twenty years before writing the foregoing. I followed the same practice in all my operations, and my results are as favorable, I am sure, as those who practise the wholesale sacrifice of the integument. Thomas, of New York, not long ago recommended a method of operation in which the tumor is enucleated without the sacrifice of any skin. Of course this is only applicable to those cases in which the skin is not already involved.

In operating upon the mammæ for scirrhus the same broad principles of surgery must be applied that are applicable to surgical interference upon any other part of the human economy. It will be of inestimable value to an operator when he has finally mastered the maxim that the principle never changes whatever the site of the disease may be. During the last few years I practised antisepsis in operating upon these cases, as I did in all my other surgical work, and I am sure the healing of the wound was so greatly accelerated that I shall never attempt the same operation without taking like precautions, and one may truly here say the end justifies the means.

The following table is an accurate account of the twenty-five successive cases which I present you:

| | DATE OF OPERATION. | NAME. | AGE. | SITE. | PERIOD OF IMMUNITY. | PERIOD OF IMMUNITY. |
|---|---|---|---|---|---|---|
| 1 | Jan. 12th, 1872. | Mrs. A. | 39 | R. | Healed nicely. No return. | No return. |
| 2 | March 3d, 1873. | Mrs. D. | 56 | L. | Healed nicely; recurred again in 1877. Died in 1882. | Four years. |
| 3 | Dec., 1873. | Mrs. A. | 47 | L. | Healed slowly; no return for five years. Lost sight of. | ——— |
| 4 | Jan. 19th, 1874. | Miss P. A. | 44 | L. | Healed nicely; perfect health for nine months; then returned and died inside of year. Died in 1875. | Nine months. |
| 5 | April 3d, 1874. | Miss May S. | 39 | L. | Very large tumor. Healed nicely. No return. | No return. |
| 6 | April 17th, 1874. | Miss Jane T. | 43 | L. | Very bad case. Insist of operation; large ulcerating surface; very weak. Recurrence in wound and died in six weeks. | None. |
| 7 | Nov. 13th, 1874. | Miss L. | 47 | L. | No recurrence. | No return. |
| 8 | Jan. 13th, 1875. | Mrs. T. | 52 | | First operation healed nicely; returned in 1876; operated again; healed again, and died early part of 1877. | 1 year and operation. 1 year and death. |
| 9 | June 10th, 1875. | Miss May S. | 36 | R. | Healed perfectly; no return for two years; left then affected. Removed it December 3d, same year. Seemed to have recovered entirely, but recurred in both breasts and died June 13th, 1877. | 2 years and operation. 3 years and death. |
| 10 | Jan. 12th, 1876. | Miss S. | 39 | L. | Recovered and no return. | No return. |
| 11 | Sept. 22d, 1876. | Mrs. P. | 48 | R. | No return. | No return. |
| 12 | Jan. 3d, 1877. | Mrs. T. | 42 | R. | No return. | No return. |
| 13 | April 7th, 1878. | Mrs. T. | 69 | R. | No return. | No return. |
| 14 | April 10th, 1879. | Mrs. D. | 45 | R. | No return. | No return. |
| 15 | Dec. 11th, 1880. | Mrs. O. | 51 | L. | Return and death in two years. | Death in two years. |
| 16 | Feb. 17th, 1881. | Miss L. | 73 | Both | Died in three days of shock. | ——— |
| 17 | Feb. 27th, 1881. | Mrs. T. | 44 | L. | No return. | No return. |
| 18 | June 18th, 1882. | Miss A. | 49 | L. | No return. | No return. |
| 19 | Aug. 7th, 1882. | Mrs. P. | 51 | L. | No return. | No return. |
| 20 | May 13th, 1883. | Mrs. K. | 53 | L. | No return. | No return. |
| 21 | Oct. 29th, 1883. | Miss D. | 31 | L. | No return. | No return. |
| 22 | July 5th, 1884. | Mrs. F. | 27 | L. | No return. | No return. |
| 23 | Oct. 3d, 1884. | Miss T. | 37 | R. | Died within one year. | ——— |
| 24 | Feb. 9th, 1885. | Miss L. | 42 | Both. | Second operation; first by some one else. Died within one year. | ——— |
| 25 | Feb. 23d, 1886. | Mrs. T. | 44 | L. | No return. | No return. |

In cases Nos. 1, 2, 3, 4, 5, 7, 8, 10, 11, 12, 13, 14, 15, 17, 18, 19, 20, 21, 23, 24, 25, there was no particular incident that calls for mention here.

In the sixth case I had little hope of ultimate recovery at time of operation, the tumor was a very large one, and presented a great ulcerating surface; it had been of three years' standing. The woman was greatly emaciated through her suffering and the attending cachexia generally. In removing it I had to extirpate a large part of the greater pectoral muscle. I then found that the minor was also involved, and that there were attachments to the ribs. I cut this away, and chiseled the involvement of the ribs away, a large part of the axellary glands where removed, but in spite of all, as soon as granulation set in it was evident there was a return of the disease, and the woman died in six weeks from the date of the operation.

In case No. 9 it was somewhat different. The first tumor occurred in the right breast in May, 1874. I operated in June, 1875, a few days after I first saw her. The wound healed perfectly, and for two years there was no symptom of any recurrence, and I had begun to hope it might be a cure, when in August, 1877, she returned to me with a nodule the size of a walnut in the left breast. I advised a second operation, to which she submitted. She once more made a good recovery, and there was nothing to indicate a recidive until early in 1880, when a tumor again appeared at the site of the original wound in the right breast, quickly followed by one in the left breast, and she finally succumbed in 1883, eight years after the original operation.

Case No. 16 is that of the old woman, previously referred to. The operation was done as quickly as practicable to avoid unnecessary shock; a continuous cut being employed in the removal of each tumor, ligating all vessels after the removal. In this way less blood is lost, and the shock is not so great as when one continually stops to apply a ligature at each bleeding point. Nevertheless she never rallied and died of shock in three days.

In case No. 22 the early age of the occurrence of the tumor is worthy of note. The patient was just a little past twenty-six years of age when I first saw her. Some eight or ten months later I advised enucleation, although I doubted scirrhus at the time. She was then operated upon, and the specimen sent to my friend, Dr. Dellafield, of New York, for examination; he pronounced it a scirrhus. This was the first case in which I practiced antisepsis. The wound healed by first intention and there has been no return.

In doing these operations asceptically the most scrupulous care must be practised. The part is first scrubbed with soap and water, then washed with ether, and then a stream of 1 to 1000 of bichloride of mercury allowed to play over it. It is then swathed in towels, which have been previously boiled and placed in the same solution for forty-eight hours; all the instruments have been asceptically rendered by prolonged boiling and immersion in strong carbolized solutions. The sponges have been prepared, and the catgut treated in chromic acid and bichloride solution, or oil of juniper and the latter. The operator and assistants practised the same scrupulous care with regard to themselves; scrubbing the hands and washing them in the mercurial solution. Iodoform is freely used in the wound, and the final dressings consist of half a dozen or more layers of gauze rendered asceptic. After the dressings are applied, and the temperature remains about the normal point, they are not touched for two weeks, when upon removal it will be found that the wound has united by first intention.

The following papers were also read and discussed:

"Cerebral Abscess." By Thos. O. Summers, Jacksonville, Fla.

"Trephining in a Case of Intermeningeal Hæmatoma with Hemiplegia." By S. S. Armstrong, Memphis, Tenn.

"Skin Grafting." By J. D. Griffith, Kansas City, Mo.

"Complete Loss of Scalp, including the greater portion of the Eyelids and one Ear. Treatment by Skin Grafts." By F. C. Shaefer, Chicago.

"Retroflexed Splints for Fractures of the Forearm." By W. D. Kearns, Pittsburgh, Pa.

"A Deformity and Disability following a Form of Injury to Ankle Joint." By E. A. Wood, Pittsburgh, Pa.

"Treatment of four cases of Acute Traumatic Tetanus," By R. C. McCord, Lebanon, Ky.

"Report of twelve cases of Alexander's Operation." By J. H. Kellogg, Battle Creek, Mich.

---

### SECTION ON MEDICAL JURISPRUDENCE.

Dr. Frank S. Billings, Director of the Patho-Biological Experiment Station of the State University of Nebraska, read a paper on "The Necessity of a Uniform Standard of Education in all American Medical Schools."

The author is directly opposed to the existing system of medical education, and finds nothing worthy of praise as it now is. The professional results that have been creditable to American medicine have been due more to influence outside the schools than to the schools themselves. The medical school system is rotten from its foundation. Its existence and purpose conflict with the true interests of the State.

It is a case of great advantages derived by the few at the expense of the many. It is more an advertising system than anything else. Doctors are made, physicians are born; doctors treat themselves to fill their own pockets, and physicians treat to save suffering humanity. The physician is the true follower of the principle that public health is public wealth; the doctor is the Iscariot of the medical profession.

The speaker believes that the greatest curse to American medicine is the "Alma Mater" nuisance, and students are used for mere business purposes, not only while students, but after having graduated. He considers this nuisance one of the main features, giving rise to dissensions among medical experts in the courts of law. The aim of the majority of American medical schools is to make doctors simply because it pays, and because those made think it will pay to become such.

In the opinion of the author not one of the American medical schools has been founded in the interest of the public alone.

The great variation in the course of instruction in the different medical schools is compared, the standard being 56 at the last annual report. The actual number of days' attendance upon lectures required varies between 18 and 621 days; this is after deducting Sundays and holidays. In Missouri, out of eight (8) regular schools, the course varies from 224 to 381 days. In New York, from 190 to 621 days.

The remedies proposed by the author for this condition of affairs are found in the following conclusions, which terminate his paper:

First—No uniformity of action can be had in forensic action until some method is devised bringing the regular medical schools into line and inaugurating one system of instruction and one system of graduation in them all.

Second—Forensic medicine must become a specialty for both medical and legal experts, both of which must take a full course in medicine and law.

Third—The desired ends can be best obtained by inaugurating a course for the medical schools in the different parts of the country by the general government, which shall support them; one system of education and one system of graduation dominating these schools. There should also be a national school of comparative medicine to be the chief point of original research and medical education in the land.

Fourth—The inauguration of a course of instruction in pathological anatomy and general pathology in all schools.

Fifth—The condemnation by the American Medical Association of all medical schools not requiring a three years' course of study of nine months each, and the abolishing of one year's study with a regular graduate.

The following papers were also read and discussed in this section:

"Medical Jurisprudence in its relation to Undue Influences as Affecting Wills and Contracts." By Judge Amos G. Hull, Néw York.

"The Suppression of the Illegal Practice of Medicine." By Joseph F. Edwards, Philadelphia, Pa.

"State Supervision of the Insane" and "Medico-Legal Relations of Epilepsy." By James G. Kiernan, Chicago, Ill.

"Mental Responsibility in Inebriety." By T. D. Crothers, Hartford, Ct.

"Syphilis and Marriage." By J. V. Shoemaker, Philadelphia, Pa.

---

OBSTETRICS AND DISEASES OF WOMEN.

Dr. Morris in his paper on "Placenta Prævia" said that three fatal cases of placenta prævia that had recently come to his notice, two of which he had seen in consultation, convinced him that some more positive and radical views of treatment should be adopted in this grave trouble than those suggested in the text books of the day.

He had not, however, prepared a lengthy paper on the subject, but in lieu thereof, had formulated some propositions which he believed would serve as incentives to discussion, and thus call forth the views and experience of the members of the Section.

Dr. Morris laid down the following propositions:

1. No expectant plan is justifiable in cases of placenta prævia. The uterus must be emptied as soon as possible after the discovery of the trouble, no matter what the stage of pregnancy may be. A halting, hesitating practice means danger both to mother and child.

2. That the life of the child must not be considered in the treatment of the case.

3. That the manner of emptying the uterus must be left to the individual judgment of the medical man in attendance.

4. That in cases of central adherence of placenta the safest and best practice is to separate the placenta entirely.

5. That in cases where the placenta is adherent in the latero-cervical zone of the uterus partial detachment may be sufficient, but if the hæmorrhage is not arrested, the whole mass should be removed and means of delivery at once instituted.

6. That the colpeurynter is the only tampon that can be safely used in these cases—that sponge, silk handkerchiefs and other forms of tampon are nasty, filthy and septic, and should never be employed.

7. That the bi-manual treatment, whenever possible, is the best and the speediest form of delivery.

8. That chloroform must be administered in all cases in which manual interference is necessary.

In discussing these propositions, Dr. Morris said that there is no emergency in obstetrics so dangerous, and in which skill, judgment and energy are so necessary, as hæmorrhage resulting from placenta prævia. Statistics show that the maternal mortality in these cases is from twenty-five to forty per cent. Of the four last cases that had come under his notice, all in consultation, three proved fatal—two from septicæmia, on the fourth day, and the third from sudden and uncontrollable hæmorrhage. The fourth case, which recovered, was treated by a pupil of his own, who had lived in the Maternity Hospital, Vienna, for two years and was thoroughly acquainted with the procedures of that school. Dr. Parvin could not have had much experience in these cases, or he would not have recommended delay or palliative measures in these cases as he has done in his recent book. Patients die from two causes, anæmia and septicæmia. Septicæmia becomes essentially fatal in the presence of anæmia. The anæmia is the result of the repeated hæmorrhage permitted to take place under the expectant plan of treatment.

Statistics show that in cases of placenta prævia two-thirds of the children perish before the full term, and of those that are born alive one-half perish soon after birth; therefore, considerations of humanity would lead us to think only of the safety of the mother; indeed, in adopting measures for her speedy delivery, without regard to the safety of the child, more children would be saved in the end.

The manner of emptying the uterus must be left, as before stated, to the individual judgment of the practitioner.

The colpeuryter is the only tampon that can be safely used. It answers many purposes; it serves to arrest hæmorrhage by the pressure it exercises; it softens the cervix and leads to gentle and gradual dilatation of the os; it relaxes the vagina and soft parts, and thus prepares the way for the use of dilators, such as Molesworth's or Barnes' bags, or even the fingers, if practicable. The colpeurynter should be filled with hot water —hot as the patient can bear; this should be renewed every hour. Cotton and sponge tampons are a nuisance and should be discarded.

Colpeurysis is, he said, little known to the general profession. It is only those gentlemen who have been educated in Germany, particularly Vienna, who are acquainted with its remarkable usefulness. There is no other means so valuable in the induction of premature labor.

The safest and best mode in cases where the placenta is centrally adherent is to separate it entirely. This arrests hæmorrhage and clears the way for further procedures looking to rapid delivery. It is claimed, it is true, that the hæmorrhage does not proceed from the placental surface supplied by the tortuous uterine arteries, but from the uterine veins. This may be true to some extent; but when the placenta is removed pressure can be brought to bear on these bleeding vessels and the hæmorrhage thus be arrested. Separation of the placenta itself furnishes a source of irritation which excites the uterus to action, but we cannot trust to the mere hæmostatic resources of nature. The cases in which there is no uterine action are the most dangerous and require the promptest measures. The manipulation necessary in this condition will generally induce contractions. Astringents and ergot are useless; they only serve to encourage delay when delay means death.

When the head presents and labor pains are active, no manual interference is necessary. In cases of this character the hæmorrhage is absolutely arrested by the pressure of the head.

The bi-manual plan of delivery, recently brought into use, is, no doubt, the best and speediest form of delivery, but it is not always practicable. The young gentlemen who have recently returned from Vienna describe it as a very easy procedure ; but I am quite sure that gentlemen educated in the old school of midwifery will not so quickly recognize its great facility. Of course, if bi-manual version is not effected, turning by the ordinary method is the only alternative.

The following additional papers were read and discussed:

" Ante-Partum Hæmorrhage. " By Wm. M. Findley, Altoona, Pa.

" Eclampsia." (Author deceased). Paper read by title. By Wm. T. Taylor, Philadelphia.

"Rupture of Uterus." By W. H. Wathen, Louisville, Ky.

" Management of Occipito-Posterior Position." By L. G. Boisliniere, St. Louis, Mo.

" Relationship between Puerperal Fever and Erysipelas in both its Acute and Dormant Forms." By A. McLaren, St. Paul, Minn.

---

### SECTION ON OPHTHALMOLOGY, OTOLOGY AND LARYNCOLOGY.

Dr. J. L. Thompson, of Indianapolis, read a paper on " Observations on Displacement of the Crystalline Lens from Congenital and Other Causes." He said:

The first three cases in the above report corroborate the writings of others as to the influence of heredity in congenital displacement of the lens.

Cases II and IV lead one to the conclusion that an eye with a congenitally displaced lens is an exceedingly weak organ, and very prone to progressive luxation, to cataract, and to glaucoma.

All of these were myopic, the myopia being due to lens anomalies and not to elongation of the eyeball—as was proved, both by the ophthalmoscope and by positive glasses, in the cases where the lens had fallen below the axis of vision.

I would also beg leave to call attention to the methods employed in replacing the lens in case No. II, and would suggest that, should one fail by manipulation, he would be justified in evacuating the anterior chamber by a free corneal incision, taking care, of course, not to wound the lens capsule, as one might by that means confidently expect the lens to bound through the pupil the moment that the aqueous escaped.

The most fruitful cause of spontaneous luxation of the lens is subacute inflammation of the uveal tract. Often have I seen it take place during the course of aquo caprulitis keratitis punctata.

Spontaneous luxation of the lens is also often the cause of acquired myopia in elderly persons. Not infrequently have I seen persons who on examination were found to be highly hyperopic and for whom I have prescribed positive glasses, even for infinite distance, and have seen these same persons again, when they needed just as strong negatives as they formerly did positives; but have almost invariably found the anterior chamber more shallow than when first examined, and congestive changes in the fundus with floating bodies in the vitreous humor. Case X shows that the spontaneous cure of cataract is by no means impossible, that it cannot always be accounted for on the theory of a falling of the lens below the upper pupillary margin, nor by injuries rupturing the capsule and subsequent absorption; but that it does, in a small number of cases, first become dislocated, then disintegerate, liquify, and regain transparency.

In luxations of the lens from traumatic causes, such peculiar and marvelous results are so often met with that were one to write a book on that subject alone, he would be classed with such authors as DeFoe or with Herodotus prior to the excavations about Nineveh and Babylon.

Resumé:

"1st. Heredity plays an important part in congenital displacement of the lens.

"2d. Congenital displacement of the lens is a dangerous anomaly which is very liable to lead to progressive luxation, to cataract and to glaucoma.

"3d. Most cases of congenital displacement are highly myopic, and said myopia is due to the malposition of the lens and not to elongation of the antero-posterior axis of the globe.

"4th. Spontaneous luxation of the lens is, in a very large proportion of cases, due to congestion and sub-acute inflammation of the uveal tract, and "second sight" as the acquired myopia of elderly persons is often due to this cause.

"5th. Partial, spontaneous luxation of the lens occasionally results in the spontaneous cure of cataract, not as is usually the case, by the falling of the lens below the upper margin of the pupil, but by degenerative metamorphosis and frequent liquefaction.

Other papers were read, as follows:

"Sympathetic Opthalmia." By C. M. Hobby, Iowa City, Iowa.

"Hydrobromate of Hyoscine as a Mydriatic." By J. M. Ray, Louisville, Ky.

"Treatment of Hypopyon Keratitus with Frequnt Irrigations of Sublimate." By F. C. Holtz, Chicago, Ill.

"Some of the Ophthalmological Clinics of Europe." By J. W. Heustis, Pittsburgh, Pa.

A boy, illustrating the results either of pemphiges or essential shrinking of the conjunctiva in both eyes, was exhibited by Robert Tilley of Chicago, Ill.

### SECTION ON DENTAL AND ORAL SURGERY.

In this section the papers announced for the afternoon's session were not read, but Dr. Charles P. Pruyn read a paper on "Dental Diagnosis and Prognosis," followed by Dr. A. E. Baldwin on "Conservative Dentistry."

### SECTION ON STATE MEDICINE.

Those in attendance upon this Section listened to the reading and discussion of the following papers:

"Influence of Military Life on the Health of the Soldier." By Major Mersi K. Taylor, Surgeon U. S. A.

"The Physical Basis of Brain Work." By Woods Hutchinson, of Iowa.

"Hygiene of Infancy and Childhood." T. B. Grenley, of Kentucky.

### SECTION ON DISEASES OF CHILDREN.

The following papers were read before the members of this Section:

"Spasm of the Larynx and Oesophagus." By C. Briggs, St. Louis, Mo.

"Infantile Feeding, especially with reference to Subjects with Infantile Eczema." By L. Duncan Bulkley, New York.

---

### SECTION ON PRACTICE OF MEDICINE, MATERIA MEDICA AND PHYSIOLOGY.

The following papers were read in this section: "Glanders in the Human Subject, with a Case." By C. N. Cooper, Cleveland, Tenn.

"Unique Case of Athetosis resulting from a Railway Shock." By C. H. Hughes, St. Louis, Mo.

"Diseases of the Dura Meter producing Motor Paralysis, Facial Spasms and Neuralgia." By Wm. B. Fletcher.

"A New Method of Producing Local Anæsthesia of the Skin." By H. J. Reynolds, Chicago.

"Aids in the Prevention of Fevers." By R. W. Seay, Fitch's Point, La.

---

### WEDNESDAY'S PROCEEDINGS.

---

### THE GENERAL SESSION.

The Association was called to order at 10 a.m. by President Gregory. After prayer by Rev. Mr. Gunsaulaus, D. D., Dr. J. V. Shoemaker moved that the rules be suspended and the business taken up and disposed of before the addresses were heard. It was unanimously carried.

The committee of arrangements gave notice "that the exhibition of the police patrol would be repeated this morning on Michigan avenue south of Jackson."

Dr. N. S. Davis asked permission to give notice that the library of the late lamented Professor Jewell was on sale at the store of A. C. McClurg & Co., corner Wabash avenue and Madison street, and the instruments, etc., at Sharp & Smith's, 73 Randolph street. He said these included the greater part of the estate, and all sales made would much benefit the children of one of the most untiring men in the medical profession.

The Board of Trustees for Publishing the Journal presented their report for the year ending March 31, 1887. The report of Dr. N. S. Davis, the editor of the Journal showed that the weekly edition had been 4,387 copies, of which 3,478 have been sent to members of the Association and 909 to regular subscribers and exchanges. This shows a net increase in the membership of 104 and in total weekly circulation of 116. There have been 4,800 copies of the Journal printed each week.

The receipts at the office of publication were: Moneys received from subscribers, advertisers, reprints, and extra journals have been $7,580.63, of which $2,494.09 was from subscribers, $751.35 from reprints and $4,335.19 from advertisements. This makes a net increase of $2,250.17 in receipts over the previous year. The cost of publication has been $13,162.01 of which $751.35 was for reprints, making the cost of the publication of the Journal $12,410.66, an increase of $1,426.99 over the preceding year. The cash value of the association printing office, after deducting 15 per cent. for wear, is $921.34 to which $137.22 worth of type, etc., have been added during the year. The actual additions to the association during the year was 506, and new subscribers 18. But 493 members have been dropped from the lists for various causes.

Dr. Wm. G. Eggleston has continued to fill the position of assistant in the editorial chair.

Only $2,758.95 has been expended during the year for editorial work.

The Journal will be increased four pages during the coming year, and Dr. Davis will continue in the editorial chair.

Dr. Deering Roberts, of Tennessee, rose to a question of privilege, on a matter in the hands of the Secretary. The Secretary read the following communication:

*To the President and Members of the American Medical Association:* — GENTLEMEN: Satisfactory acknowledgments having been made in *The Chicago Times* of this morning as regards its assertions in the editorial columns of a preceding issue, reflecting upon the regular medical profession of Chicago and elsewhere, I would most respectfully beg leave to withdraw the resolutions I offered on yesterday morning. Very respectfully, etc.

DEERING J. ROBERTS, M. D.

Dr. Morris, of Baltimore, moved that Dr. Roberts be allowed to take his resolutions from the table. Carried.

Dr. N. S. Davis read the report of the Special Committee on Changes in the plan of Organizations and By-Laws of the Association, appointed in accordance with the following resolution adopted by the Association at the last annual meeting:

*Resolved*, That a committee of nine members, including the President, the President-elect and four Vice-Presidents elect, be appointed by the Chair to consider the various propositions looking to the amendment of the organic law of the Association by the establishment of branches or in any other way; said committee to report at the next annual meeting what measure of organization, if any, may be desirable."

This resolution was inspired by the desirability of a more permanent and representative business committee to perform the work done by the nominating committee, and, second, the desirability of increasing the paying permanent membership. Some had suggested the formation of branches, the members of which would be permanent members of the Association and therefore liable for annual dues. This plan, copied after the British Medical Association, the Committee did not favor.

Dr. Rayburn moved that the report be received.

Dr. Jennings moved that the report be received and adopted.

Dr. Wathen saw objections to the report, but nevertheless hoped it would be adopted.

The motion was put as amended and carried.

Then followed a long and animated discussion as to the legality of adopting the amendments to the constitution without their laying over for one year.

Dr. N. S. Davis made the point that, under the resolutions appointing the committee, the matter could possibly be constituted as an amendment.

Dr. Quinley thought the whole proceeding out of order. The one way was to adopt the amendments to the by-laws and allow the amendments to the constitution to lie over.

Dr. Bell moved that it was the sense of the Association that the amendments had been presented a year ago.

After an animated discussion, during which much confusion existed, and the chair appeared puzzled, a vote was taken on the motion of Dr. Bell, which resulted in 272 for the motion, and 232 against.

On the announcement of this decision there was much confusion, and comment was vigorous. The chair ruled all out of order and proceeded with the business; the next being the call of the nominating committee which was as follows:

NOMINATING COMMITTEE.

Alabama, W. C. Cross; Arkansas, C. G. Ewing; California, J. W. Robertson; Colorado, C. Drummond; Connecticut, W. H. Whitmore; District of Columbia, J. M. Toner: Delaware, ——; Florida, M. B. Phillips; Georgia, A. G. Whitehead; Illinois, E. P. Cook; Indiana, T. B. Harvey; Iowa, Wm. Watson; Kansas, W. L. Skenck; Kentucky, D. S. Reynolds; Louisiana, T. G. Richardson; Maine, D. E. Marston; Maryland, T. B. Evans; Massachusetts, E. W. Cushing; Michigan, Wm. Brodie; Minnesota, J. A. McGaughey; Mississippi, T. R. Trotter; Missouri, J. M. Allen; Nebraska, W. M. Knapp; North Carolina, Eugene Grissom; New Hampshire, J. W. Parsons; New Jersey, Lott Southard; New York, Darwin Colvin; Ohio, X. C. Scott; Oregon, ——; Pennsylvania, E. A. Wood; Rhode Island, Wm. J. Burge; South Carolina, Thomas Legare; Tennessee, J. B. Murfee; Texas, R. W. Park; Vermont, S. H. Griswold; Virginia, Louis M. Nash; West Virginia, ——; Wisconsin, J. K. Bartlett; United Army, ——; United States Navy, D. Bloodgood; United States Marine Hospital Service, H. M. Goldsboro; Dakota, E. M. Darrow; New Mexico, Russell Bailey.

The meeting-place for the Nominating Committee was fixed for Apollo Hall.

The Secretary announced that the American Pharmaceutical Convention had appointed a Committee to present resolutions to the American Medical Association.

The next order of business being the reports of Chairmen, Dr. J. B. Lynch read the report on The Practice of Medicine, after which the Society adjourned to Thursday at 10 a.m.

---

SECTION OF ORAL AND DENTAL SURGERY.

The Section was presided over by Dr. Marshall, the Chairman.

The first paper was by Dr. K. B. Davis on "Pathological Conditions of the Teeth and Their Systemic Effects."

Dr. G. Frank Lydston, of Chicago, then read a paper entitled: "The Co-relation of General and Dental Medicine and Surgery." As the subject is one on which there is a wide divergence of opinion among dentists, we publish rather full abstracts. Dr. Lydston said:

Having been invited to address you upon some topic of a character suitable for presentation to progressive dentists and oral surgeons, I have thought it advisable to present to you my reasons for believing that the interests of the general practitioner and dentist have much in common. Personally, I am greatly interested in dentistry as a department of the great science and art of general medicine, and in my association with dentists I have derived both social and professional benefit, and have come to regard them as the co-laborers of their medical and surgical brethren in the field of science.

It was long argued by the members of my own department of science that the normal position of the dentist is that of a species of tooth carpenter—a mere mechanical drawer of teeth and plugger of real or imaginary cavities; and it is a serious reflection upon the liberality of the medical profession that many of its members accord much the same position to the dentist of to-day.

In not a few instances the modern surgeon had indignantly resented the claims of dentistry as a surgical specialty, apparently forgetting in his illiberality that it was only about a hundred years ago that the general surgeon himself began to rise above the level of the barber and the monkish cutter for "the stone." Had there not been a Hunter, who arose like an apostle of scientific light, and began his physio-chirugical experiments years in advance of his day and age, the present status of general surgery might be most humiliating to contemplate.

It is an amusing fact that in some of our latter-day illustrations of the crudlity of the surgery of a past generation, dentistry and general surgery are most intimately associated. How consoling and gratifying to the conceit of ye pompous surgeon of to-day is the legend still to be occasionally seen surmounting a barber pole: "Bleeding, leeching, cupping, blistering, tooth-drawing and hair cutting done here."

Wedded centuries ago, Surgery and Dentistry drifted away from the barber, and, unfortunately, also from each other, pari passu with scientific progress. In these modern times we are striving to reunite the two branches of healing by a common interest in science, and upon the basis of a division of labor, i. e.: specialism. It is perhaps fortunate for all concerned that an occasional barber exists who is capable of demonstrating to us the common and humble origin of both specialties.

There has been a striking interdependence of most of the modern improvements in dental and surgical science. Although not generally so accepted, John Hunter's famous experiment of grafting the spur of a chicken cock upon its comb really foreshadowed not only the operation of skin grafting, but that of tooth grafting and tooth implantation, a procedure which has recently been brought so prominently before the dental profession by Dr. Younger. It is indeed singular that the operation of tooth transplantation and implantation was not earlier discovered, inasmuch as the various epithelial tissues and appendages are much alike in their manner of growth and development, and the growth of an implanted or transplanted tooth is hardly more remarkable than the phenomena attendant upon the attachment and growth of miliary skin grafts. That a tooth which has been apparently dead for a considerable time should grow after implantation is no more wonderful than the growth of skin grafts which have been transplanted from the dead to the living body. I have myself succeeded in the transplantation of grafts from a corpse dead forty hours, to a healthy living ulcer. As an absolute verification of this, I have grafted the skin of a negro, dead nearly thirty-six hours to the leg of a white man, and with success, the area of black skin resulting being quite conclusive. Another peculiar fact is that apparently effete epthelium scraped from the surface of the skin, and sprinkled over the granulations of a healthy ulcer, will result in the development of new little islets of skin and thus materially hasten cicatrization.

I understand that living teeth have been transferred from the human mouth to the comb of a cock, and thus preserved for future use, the teeth meantime absorbing nutriment from the circulation of the fowl. If this be practicable, the procedure brings dental surgery of to-day and the now famous experiment of the immortal Hunter very near each other. While my digression may seem irrelevant to some, I think that it is justified by this kinship between skin grafting and tooth grafting.

The histo-genetic power shown to exist in epithelial structures has been shown to exist in others. There seems to be a general law pervading all organic life, to the effect that the power of reproduction and repair of organized matter is in inverse ratio to its degree of differentiation. This holds true of cells as well as bodies completely organized. The star-fish may be dismembered, but it reproduces the lost segments. Smash the amæba, and we have as many new amaeba as we have particles. Spiders, crabs, craw fishes, and some other animals speedily reproduce lost limbs and other appendages. So we see in cells, that epithelial cells are possessed of great vitality, and not only grow themselves, but excite the property of rapid growth in any cell with which they may come in contact. Degraded epithelial cells grow more rapidly than the more perfect forms, as is evidenced by those sad cases of carcinoma and sarcoma so often met with. Connective tissue cells have an innate power of proliferation and propensity for differentiation upon which all processes of repair and growth of all kinds, physiological or pathological, depend. Irritate the tissues, and these propensities are forthwith brought into action.

The labors of the general surgeon showed us long ago that bone could be reproduced from periosteum, and that most daring operator, the late James R. Wood, showed in his noted case of phosphorus necrosis, that the entire lower jaw might be reproduced after its ex-section.

The dental and oral surgeon has not been at all backward in taking advantage of this extraordinary reproductive power of the maxillary periosteum, and my progressive friend, Dr. J. S. Marshall, has recently operated upon the maxillary in a manner which would put some of oui general surgeons to the blush. Such operations do not savor very strongly of the tooth-pulling mechanic.

The dentist has not only shown himself to be capable of assimilating the discoveries of surgical science, but he has contributed materially to the art of surgery. To a dentist we owe the discovery of anæsthesia, the greatest boon ever conferred upon suffering humanity.

Much of our knowledge of that valuable drug the peroxide of hydrogen has been due to its use in the practice of dentistry.

Even the field of microscopy has been invaded by the dental scientist. Much of our present knowledge of the forms and growth of micro-organisms is due to the researches of that indefatigable worker Dr. G. V. Black; and it would be well if every physician would study the results of this gentleman's labors in his chosen field.

We have not only come to regard dentistry as a special branch of the healing art, but we are forced to acknowledge that it is a most important branch.

In the words of the late Professor Gross, "Dentistry is the most important specialty in medicine. Many people come into the world and go out of it who never require the services of other specialists, but no child is born that does not sooner or later require the services of the dentist." The words of the Nestor of American Surgery are excellent testimony in favor of dentistry as a necessary specialty, and it would be well if this truth were more generally appreciated.

The classification of dentistry as a specialty of general surgery is justified to a greater degree than in the case of any other specialty. As my lamented friend, the late Professor Van Buren, used to define it, "Surgery is that branch of the healing art which takes charge of all those diseases and injuries which require in their management the use of instruments and mechanical appliances, operations, or especial manual dexterity." To no other specialty does this definition so aptly apply as to dentistry, and I can scarcely conceive of a single dental or oral operation that does not require all of the qualifications mentioned. This eminent surgeon said further, with reference to general surgery as a specialty of medical science, "that a specialty should be the outgrowth of a liberal professional education"; in short, that a true specialist should be a man who knows "something of everything, and everything of something." Every specialty should be evolved from the general field of scientific labor, and its selection should be modified by experience, the law of division of labor, and special adaptation. In the present paper we have but to do with the dental specialist, and it is to him in particular that we at present apply this general rule.

The dentist is nowadays expected to be a fair anatomist, and it should no longer be considered reprehensible for him to know more than the anatomy of the teeth and jaws. He may never realize the fact that he has an ileo-cæcal valve, or a vermiform appendix, but a little enlightenment upon the subject will not render him less capable of skillful dental work; and when an obstreperous cherry pit lodges in his appendix, he may console himself in the fact that he knows pretty nearly where the trouble is located. He is, of course, expected to know all about the structure and development of the teeth and jaws, the muscular mechanics in their movements, and their nervous and vascular supply, in order that he may occasionally discourse learnedly upon centers of classification, intermaxillary bones and synchondroses *ad infinitum*, as does my enthusiastic friend, Dr. Talbot, when discussing his hobby, "dental irregularities." The necessity for a knowledge of a physiology is self-evident, the embryonal development and eruption of the teeth being of especial importance. A knowledge of the physiological secretions is a necessity, else how could the effects upon the teeth of the morbid conditions affecting them be studied? In the study of normal and morbid secretions a knowledge of chemistry is necessarily involved. Chemistry and metallurgy are further necessary, in order that the student of dental science may become familiar with the various materials and implements with which he works and the composition of the teeth. The dentist requires a certain amount of therapeutical and pharmaceutical information. The dental *materia medica* is becoming an important factor in dental education.

Perhaps the most important branch in dental education is pathology and pathological anatomy, particularly that division of the subject which has been termed "surgical pathology." The amount of general medical and surgical knowledge requisite to a clear understanding of the morbid changes which are under the attention of the dentist is far greater than is supposed by those narrow-minded individuals who regard dentistry as a species of tooth carpentry. The dentist must thoroughly understand the forms, causes and treatment of caries and necrosis of the teeth and jaws, a knowledge of which will open up to him a vast field of general surgical information. If he understands maxillary necrosis he will also understand the same disease as affecting other bones. He should pay considerable attention to syphilis and its treatment, as causes of caries and necrosis of the teeth and jaws, and inflammation or ulceration of the mucous membrane of the mouth. Being familiar with such lesions, he must necessarily know much of the mucous inflammation and ulceration in other situations. He must learn all about the causes, anatomy and treatment of suppurative inflammation about the jaws, and when he has done so he will have mastered the most important portion of general surgery.

Neoplasmata, their causes, method of formation and structure, should be familiar to him, for he may be confronted at any time with epulis cancer of the jaws, extosed or other conditions requiring surgical skill for their relief or cure. He should know all of the important details regarding fractures, their method of union, and mechanical and therapeutical treatment.

The necessity of a thorough uuderstanding of the general principles of medicine, particularly as regards the causes, results and therapeutics of perversions of general nutrition, can scarcely be overestimated. This is most aptly illustrated in those conditions of malnutrition which result from syphilis, rickets and struma, diseases which react most injuriously upon the teeth and jaws. A fair ability to differentiate the various oral lesions of syphilis from benignant affections, and from each other, is requisite. As my views upon this subject were presented before your section at the St. Louis meeting of last year, it is unnecessary at this time to consider the topic in detail, although I consider it one of the most important that can be brought before the progressive dentist.

It is a long way around from defective molars to perverted nutrition, but from imperfect mastication may result imperfect digestion and assimilation, and from the latter condition there may occur such a strain upon the liver and kidneys that actual structural diseases result. Gout is a dyscrasic affection, which may be expected from such conditions of perverted physiology. Conversely, as has been already indicated, morbid appearances of the teeth and gums may indicate certain morbid conditions of the general system, which bear a casual relation to the dental imperfections. As illustrations may be mentioned syphilis, rickets, mercuriolism, struma and scurvy.

A subject with which every dentist should be more or less familiar is the serious affection known as septicæmia, or preferably septæmia or blood poisoning.

Instances of fatal blood poisoning have been known to follow so simple an affection as alveolar abscess, as well as more grave forms of suppurative inflammation about the jaws and teeth. Mild cases are more frequent than is generally supposed. Only a short time since I met with a case of mild septæmia following an alveolar abscess in one of my personal friends. In this instance the dentist in attendance, and a physician to whom he referred the patient prior to his visit to me, failed to recognize the real condition present.

In connection with this condition of sepsis the dentist may simplify this study of the subject if he will remember that septicæmia and pyæmia so-called, are but phases of the same constitutional condition, and are due to the local absorbtion and subsequent general dissemination of the same septic material. The differcnce between the two phases of disease are those of degree, not kind, and they depend, not upon the existence of a different materies morbi, but upon: 1st, the primary intensity of the poison; 2d, the facility with which it is absorbed; 3d, the quantity absorbed and the duration of the period of its absorbtion; 4th, the rapidity of its elimination; 5th, and the most important consideration of all, the inherent vitality or resisting power of the patient.

By studying the subject of blood poisoning in this manner, it may be reduced to a simple and logical basis. As presented in most of the works on surgery, septicæmia and its various phases constitutes a subject so confusing that very few students ever succeed in mastering its intricacies. The dental specialist should not only familiarize himself with the clinical features of septic iufection, but he should study the results obtained by the investigations of Pasteur, which foreshadowed all of our knowledge of septic processes. He should also understand the principles laid down by Lister in his system of anti-septic treatment, which involves all of those cardinal principles which govern us in in the prophylaxis and treatment of blood infection from suppurating wounds.

Look where we may in the chosen field of the dentist, and we are confronted by intricate problems of a general and most diverse surgical character. Who among our general surgeons can say after such a survey of the science and art of dental and oral surgery, that the skillful and studious practitioner of this specialty is not a surgical scientist and a co-laborer in that beneficient art whose progress has been illumined by Hunter, Bilbroth, Paget, Parn, Mott, Van Buren, Wood and others, whose names, though equally famous, would fill my paper to overflowing. I maintain that no man of even average intelligence can become a thorough dentist without at the same time becoming a pretty good physician and surgeon. He should, by all means, possess the general knowledge of the average practitioner of general medicine, and in addition, he should have the general knowledge and mechanical talent necessary to treat the peculiar class of cases brought before him as a surgical specialist. There is a special necessity for general medical knowledge in a certain direction, to which I desire to call attention. Every educated dentist is now supposed to familiarize himself with anæsthetics and their method of administration, but, unfortunately, he is not expected to know very much about those conditions which contraindicate their use. There are various morbid conditions of the heart, lungs and kidneys, which the merest tyro might discover by a little attention, and which I fear, often escape the attention of the dentist. I believe that no one should ever give an anæsthetic who is not qualified to make a fairly accurate estimate of the condition of the heart, lungs and kidneys; yet this is often done in the dentist's chair. Much can be determined by careful observation of the patient's general appearance. I recall several cases in point: A friend of mine and a competent dentist, undertook the administration of chloroform for the purpose of extracting a carious tooth. He was assisted by a lady physician who considered herself well qualified. The affair did not concern me, but as I had an adjoining office I happened to get a glimpse of the patient's face, and decided to remain within call. Hardly had the amæsthetic fairly begin its work before the stertorous breathing of the patient alarmed my friend so that he called me to his assistance. It was high time, for if ever a patient was on the verge of death from asphyxia and heart paralysis, this one was. I succeeded in recuscitating her, but she subsequently passed through an attack of renal and pulmonary congestion that well nigh ended her life. The puffy eye-lids, red face and feeble circulation of this patient should have warned my friend of the danger of vasa motor paresis to which this patient was exposed, and should have suggested an urinalysis which would have developed the existence of chronic Bright's disease. I have since seen a similar case from nitrous oxide. In this case also, there was the sequence of acute pulmonary congestion, but in a patient who was phthisical. There is an old adage to the effect that "it is a poor rule that does not work both ways." The doctor should have some special dental knowledge. Some time ago I was consulted by a young man with a suppurative tumor of the jaw. He had been treated for some weeks by an aspiring surgeon who pronounced the case one of "actinomycosis" and proposed to operate upon it. I discovered that the tumor was a phlegmonous inflammation secondary to a carious tooth, and which had existed for eight months. Dr. Austin, who saw the case with me, extracted the tooth to which I attributed the trouble, and in two weeks without further treatment, the case was well. Some of my friends who are present will recall this case, and the controversy in which I became involved on account of my inability to diagnose "the second case of actinomycosis ever seen in America."

As an offset to this case, I once saw a case which a dentist referred to me, for the purpose of having a tumor of the gum removed. I found a spongy, vascular growth the size of a hickory nut protruding upon both the anterior and posterior surfaces of the lower incisor teeth. On examination I discovered the tumor to be a mass of spongy and exuberant granulations due to the irritation produced by a mass of tartar about the incisors. Removal of the tartar with the application of nitrate of silver to the tumor cured the case completely.

A fact recently determined, and one which is of great importance, is the association of neuralgias of various kinds, and perturbations of sight and hearing, with carious teeth. This necessitates a certain amount of neuralogical knowledge on the part of the dentist.

In my presentation of the claims of dentistry as a surgical specialty, I do not wish to be understood as advocating the presumption of the rights and duties of the general practitioner by the dentist. A true specialist should render unto Cæsar those things which are Cæsar's—"i. e.: he must on proper occasion consult the general practitioner. I know of one individual who apparently attempts to monopolize the entire field of medicine and surgery. So many and varied are the cases which he collects for the gaze of the admiring public, that his office strongly reminds one of a dime museum representation of a chamber of horrors. Until quite recently this professional anomaly occupied the position of examiner to a life insurance organization. Imagine the sudden metamorphosis of the filler of teeth into the analyst of the urine. Versatility of talents is an admirable quality, but under certain circumstances its exhibition may be in decided violation of decency and good taste, to say nothing of the question of "loaves and fishes."

In passing, I will merely allude to the necessity for a knowledge of some of the natural sequences of pregnancy. The better class of dentists are of course familiar with the fact that dental operations are not always attended by good results, and are sometimes absolutely dangerous in the pregnant state. This fact, although disputed by some, is of sufficient importance to merit most serious consideration. Even if disputed, the patient and not the operation, should have the benefit of the doubt.

To show, in conclusion, that I am not alone in my desire to see the profession of dentistry recognized as a part of general medicine and surgery, I may cite the existence of the dental section of the American Medical Association, present here to-day. More pertinent still, is the fact of the establishment of a dental section in the coming International Medical Congress. It is to be hoped that the dental profession will give this department of the Congress their most earnest and harmonious support, for, if it be successful, it will give the claims of the dental profession to surgical specialism an impetus that nothing else would afford.

The third paper on the programme was one by Dr. A. H. Thompson on "Pathological Heredity and Congenital Abnormalities of the Teeth."

In this section the work has been thorough, largely due to the efficient manner in which the officers have performed their work.

The nominees for the ensuing year are Dr. J. Taft, of Cincinnati, for chairman, and Dr. E. S. Talbot, of Chicago, Secretary.

---

### SECTION ON OBSTETRICS AND DISEASES OF WOMEN.

The nomination of officers for the ensuing year were Eli Van DeWarker, of Syracuse, N. Y., chairman, and E. W. Cnshing, of Boston, secretary.

Dr. George F. French, of Minneapolis, then read a paper on

### THE CHIEF SOURCE OF DANGER IN THE USE OF THE UTERINE SOUND, in which he said:

It is the prevailing opinion of the medical profession that the principal danger to guard against in probing or sounding the uterus is traumatic lesion from incautious handling of the instrument. This is also the teaching, positively or negatively, of nearly all the leading books on gynæcology, including Emmet, Thomas, Goodell, Mundé, Hewett and Courty.

Thus, Emmet says in the third edition of his "Principles and Practice of Gynæcology:" "Many a poor woman has endured years of bad health from the carelessness of her physician in overlooking a latent cellulitis which became rekindled by the unskillful use of the probe or sound." In conformity with this view, Prof. Dudley, in his recent contribution to "Pepper's System of Medicine," conveys at once his own opinion on the subject, as well as his impression of Emmet's teaching that traumatism is the danger to be averted, by quoting Emmet as to the dangerous use of the sound "from frequent lighting and relighting of pelvic inflammations by injudicious slight manipulations."

Writing on the same subject, Mundé says, "The precautions to be observed in using the uterine sound or probe are chiefly comprised in the two words, delicacy and gentleness."

So Graily Hewitt—fourth edition, "The Diseases of Women," writes, "As a general rule patients experience no inconvenience from the use of the sound if it be carefully introduced."

In the fifth edition of Thomas, Nonat and Scanzoni are cited to show that a far greater danger attends the use of the sound than is commonly recognized. From the context the reader would infer that the danger is from traumatic lesion, at least no other source of danger is referred to. Chrobak, in "Billroth's Handbook of Diseases of Women," in citing Broca's case of death following the use of the uterine sound, says, "the number of this class of cases published and unpublished is quite large, and the number still larger where a slight or severe illness ensues without a fatal result." When we consider the remarkable tolerance of the uterus of such surgical procedures as curetting, divulsing—not to mention accidental perforation—it seems that some more obvious cause must be sought. This prolific source of danger—and one comparatively unrecognized—lies in septic matter conveyed into the uterine cavity by the non-disinfected sound; a danger, which when we come to study the histological nature of the uterine cavity will be recognized beyond a doubt. We can all remember when traumatism was the bug-bear that deterred us from entering the peritoneal cavity; and when a healthy patient every now and then succumbed to cystitis (as the death-certificate read) after being merely catheterized, we regarded it as due to traumatic shock, but we have learned better and the rationale of a septic infection is now painfully easy to understand. Some surgeons, like Mundé and Tait, are so absolutely clean in their methods of doing surgical work, no septic results occur, though they are so unconscious of septic danger as to ignore its existence in their writings. Thus Mundé sounded the uterus 5000 times without a catastrophe, which only proves that Mundé practises better than he preaches; a criticism equally true of the pyo-salpingist of Birmingham. I would not detract one iota from the importance of delicate manipulation in exploring the uterine cavity, but I maintain that the danger from traumatic lesion is completely overshadowed by that from septic infection.

Noggerath's doctrine of latent gonorrhœa is just beginning to be fully recognized, but instead of being an isolated, exceptional fact, it is one of a large group of facts which illustrate a general principle. From the researches of D. Berry Hart, Lindgren and Leopold, it has been demonstrated that the intra-uterine mucous membrane is really a lymphatic gland or a lymphatic surface intersected with glands and blood-vessels. To Hart belongs the distinction of being the first among English-speaking writers to systematically call attention to this comparatively unrecognized danger. He says: "The great dangers to the patient from the passage of the uterine sound are abortion and abrasion of the mucous membrane with absorption of septic matter and resulting cellulitis and peritonitis. Two cases of this nature are reported by Saenger of Leipsic. Manifestly a gonorrhœal pyo-salpinx might be communicated as readily by an infected sound as by the extension of the disease from the vagina. So to a septic pyo-salpinx-non-gonorrhœal, whether derived from the virus of erysipelis, pyæmia, diphtheria or scarlet-fever may be carelessly transferred from the gynæcologist's hand to the sound, and give rise to a pelveo-peritonitis with more or less systemic infection.

Among the German authors, Fritsch has done signal service to emphasize these dangers, and forcibly says: "Always before introducing the uterine sound, dip it into a three or five per cent. solution of carbolic acid, even though properly cleansed directly after its previous use."

Although these words belong to the New Testament of medical belief, and were uttered several years ago, the momentous truth inculcated is as slow of appreciative recognition as the oracles of the inspired Semmelweis.

Dr. Hiram Carson, of Maple Hill, Penn., read a paper on

ABORTION, stating it was his practice when there was hope of saving the embryo to rely on the use of anodynes to relieve pain and prevent contraction of the womb, but when he believed there was no hope of saving it and miscarriage would occur, he uses the sponge tampon in the vagina to prevent excessive flooding, and opium for its effect upon the nervous system, and then waited, sometimes months, until the mouth of the womb should open sufficiently to enable the removing of the ovum with the fingers.

He believes early dilation by tents and forceps to be dangerous, and never uses them, while with the treatment spoken of he has never lost a patient or known of any other physician losing one.

He has never used antiseptics and does not believe there is danger of septicemia from absorption of putrid blood. Deaths not infrequently occur from the use of tents, causing metritis and peritonitis, and these can not be attributed to absorption of putrid blood.

He thinks the washing out of the vagina and womb with antiseptic fluid after the miscarriage unnecessary, annoying and sometimes harmful to the patient.

The author stated the principal object of his paper was to prevent the meddling with abortion cases that is likely to result from the teachings of the day, especially the use of tents and forceps, which have proved dangerous to the mother.

Other papers in this section were as follows :

"A Case of Tubal Pregnancy, with Specimen.' By C. R. Reed, Middleport, Ohio.

"Uterine Subinvolation and Aereolar Hyperplasia." By Joseph Eastman, Indianapolis, Ind.

"A Case of Pyosalpinx—Recovery withou Operation. Remarks upon Vaginal Temperament in Pelvis Inflamation." By W. W. Potter, Buffalo, N. Y.

"Laparatomy as a Cure for Tuberculosis o the Peritoneum." By Ely Van De Warker, Syracuse, N. Y.

"Hysteria and Ovaries." By B. E. Hadra, Austin, Texas.

"Technique of Ovariotomy." By C. M. Wilson, Philadelphia, Pa.

"The Use of the Buried Continuous Animal Suture in Laparotomy and in Perineorraphy." By E. W. Cushing, Boston, Mass.

"New Form of Suture Pin for Use in Perineal Operations." By J. H. Kellogg, Battle Creek, Michigan.

---

SECTION ON SURGERY AND ANATOMY.

It was first moved and seconded that the section meet at 2 p. m. The nominations for officers resulted in the selection of Dr. Donald McLean for Chairman, and Dr. B. A. Watson, of Jersey City, for Secretary.

The first paper was by Dr. Andrews, of Chicago, on "Solution of Necrosed Bone in Spinal Abscess by Dilute Hydrochloric Acid."

Dr. H. O. Walker, of Detroit, Mich., read a paper upon "External Perineal Urethrotomy," reporting eight cases, in each combining with it internal urethrotomy.

Of the eighteen cases, reported one was operated upon twice, making nineteen operations in all. One died within a week following the operation, due to surgical kidney. Fifteen were relieved entirely of all previous symtoms, and the remaining two were much improved, although some urinary distress still remains.

In all the cases there is a tendency to recontraction of the urethra, though not from a want of thorough division of the cicatrical tisssue, as this was done in each case with the greatest care, and following out faithfully afterwards the introduction of full-sized steel sounds at stated intervals.

He also exhibited several instruments which he had devised and had made for him by Tiemann & Co., of New York. The first was a meatatome (Figure 1), which presents at its extremity a rectangular cutting edge, with a probe point at its upper border.

Tht doctor deems the gradual stretching of the prostatic urethra a proceeding of importance, not only in exploring the bladder, but in temporarily paralyzing that portion of the urethra, as it aids materially in relieving any cystitis that may and usually does exist in long standing cases of stricture of the deep urethra. For this purpose he devised the prostatic dilator as seen in Figure 4.

Figure 2 is a urethrotamy staff, to which is attached a whalebone filiform bougie, rendering its introduction easy and faciltates the completion of the perineal section with the triangular probe-pointed knife, as seen in Figure 3.

The instrument, as represented in Figure 5, consists of two parallel steel bars, the lower segmented near its distal end, and is held together by pivoted bars which can be separated or closed by means of a button screw. In the upper bar is a grove into which glides the knife, and when not in use is concealed by a small half bulb opposite the commencement of the curve. The distal used is two milimeters in diameter and tunneled,

[Figure 1.]

[Figure 2.]

[Figure 3.]

[Figure 4.]

[Figure 5.]

permitting it to glide over a guide, and by this manner we are able to cut the way through by pushing the knife forward from its concealment, and when it has passed the screw, dilating the urethra to the extent desired, that to be noted by the indicator, when the knife is drawn from behind forward and the cicatricial tissue divided. It will be seen that the instrument combines the properties of a tunneled sound, a dilator and a urethrotome.

In conclusion the doctor states that the accepted methods of treating strictures are by dilitation and incision, and that dilatation stands pre-eminent as a means of treating strictures in the great majority of cases. Yet it cannot be denied tnat the cutting strictures of large calibre has proved beneficial in many instances, and relieved many reflex troubles that would not yield to gradual dilatation. This is especially true of all strictures at or near the meatus. Although there is much weight of testimony in favor of the radical cure of stricture by incision, I am not as yet prepared to accept the statement, for I have cut many strictures dating back for a period of twenty years, and I do not know of any but will close up if not kept open by the occasional introduction of a sound. Old and tight strictures, that will not succumb to dilatation, are best treated by incision.

Dr. Robert Newman, of New York, gave a "Synopsis of a second hundred cases of Stricture of the Urethra treated by electrolysis." Dr. A. H. Wilson read a paper on "Prostate Gland; a review of its anatomy, pathology and treatment."

The discussion of these three papers was very animated, the bulk of opinion being in favor of the methods of Dr. Newman.

The paper on "Surgical Treatment of Suppurative Pleuritis in Children" by A. G. P. Garnett was read by Surgeon General J. B. Hamilton.

The author stated that it was his purpose to consider the comparative merits of the operation of removing the pus by the old method of the trocar and drainage tube, with the more modern one of resecting or excising a portion of one or more ribs, restricting this comparison to cases occurring in children. The operation for the removal of pleuritic effusion, whether of pus, serum or blood, was practiced by the ancients as far back as the time of the Hippocrates, but not until 1665 was the ordinary trocar introduced and draining the pleural sac by this means resorted to. Scultetus, one of the most ingenious surgeons of his day, was the first to use a drainage tube.

But, following the history of this instrument to the present half century, we find the late Professor Gross says that in using the trocar in tapping the chest in empyema, out of 820 recorded cases but one death occurred from hemorrhage. Of 498 cases reported by Gunther in 1861, 302 were cured, 149 died and 16 were improved; and in 1879 Dr. Blake, of Boston, reported 18 cases operated on by incision as a means of introducing the tube, with 15 recoveries. It is to be regretted these cases were not classified according to age to show the relative rate of mortality between adults and children.

It is fair to suppose the majority of the cases reported were those of adults, and if such gratifying results were obtained with the advanced and chronic condition of the disease in the adult, how much more gratifying would have been the results had the operations been confined to children.

The author presented a statistical table, prepared by Dr. Godlee, of 34 cases of empyenia in children treated at the Eastern Hospital, London, including four cases treated at Brompton Hospital, giving the age of the patient and result of operation. Briefly, it appeared that of the 31 cases reported as cured in the table, 25 were subjected to the antecedent operation of aspiration, and six primary resections. Four of the 25 were cured by one aspiration alone, and the remaining 21 all subsequently treated by removal of one or more ribs.

Had the plan of inserting and retaining the drainage tube been adopted at first it is more than probable not one of the 21 children would have been subjected to the operation of incision of the ribs.

The author quoted the experiences of Dr. Henry L. Bowditch, of Boston, as supporting his views, this gentleman having performed the operation of tapping the chest oftener, perhaps, than any other man now living.

He says he has never deemed exsection of the rib necessary in children, and with his experience his judgment on this point should be regarded as conclusive.

Excision of a portion of one or more ribs for empyema as a substitute for, or supplementary to, the operation is comparatively modern date, Dr. Warren Stone, of New Orleans, being the first to perform the operation in this country.

The criticisms presented had reference to operation on young children exclusively, as the author desired to call attention to the importance of discriminating carefully in the surgical treatment of these cases between adults and children. He presented several cases as illustrating his vlews.

### SECTION ON THE DISEASES OF WOMEN AND CHILDREN.

The following papers were read:

1. Epidermic and Hypodermic Medication of Infants, by J. A. Larrabee, of Louisville, Ky.

2. Scarlet Fever, by Dr. J. N. Love, of St. Louis.

3. Entero-Colitis, by S. E. Spern, of Delafield, Wis.

4. Infant Feeding, especially with reference to subjects with infantile eczema, by C. Duncan Bulkley. of New York.

5. "The Cause and Treatment of Infantile Eczema," by J. V. Shoemaker, of Philadelphia.

The author stated this to be one of the most common diseases of early life, and, while always disturbing and frequently obstinate, was more amenable to treatment than the eczema of adults.

It may appear in a variety of forms, and may attack all of the integument, but most frequently attacks the face, scalp, neck, chest, buttocks and upper and lower extremities.

Infantile eczema is practically due to one of four causes; first, insufficient or improper food; second, imperfect assimilation; third, deficient excretion; fourth, external irritation. The author then gave same valuable advice as to treatment.

The following formula was found valuable in connection with a liberal diet by the mother.

If the infant cannot be nursed by its mother, the best substitute is pure cow's milk, unmixed with any other substance whatever.

Cases due to imperfect digestion and ural-assimilation require careful study. Change of air is sometimes beneficial, cod liver oil, syrup of the iodiate of iron and quinine were effective remedies, the author citing when each was of most value.

In the local treatment, where itching is a marked symptom, various soothing ointments may be used, and applications of cold water, vinegar and water, leadwater and laudanum, or a saturated solution of bi-carbonate of soda will be found grateful and calmative.

Where the disease has become chronic the affected region should be covered with a starch poultice, or with oil, to loosen the crust, which must be carefully peeled off. Then stimulating ointments can be used.

The author stated he had intentionally omitted stating anything regarding the use of arsenic in the treatment. Arsenic was occasionally requisite in treating obstinate forms of eczema in adults, but was positively injurious to children. He had discarded its use in treating children for nine years, and was delighted with the results.

6. Diarrhœa Infantum, By George Wheeler Jones, of Danville.

The nominations in this section were: For Chairman, Dr. F. E. Waxham, of Chicago; and Dr. W. B. Lawrence, of Batesville, Arkansas, for Secretary.

---

### SECTION ON PRACTICE OF MEDICINE.

In this section the following programme was carried out:

1. The Treatment of Felon without Incision. By L. Duncan Bulkley, New York.

2. Antipyrin in Rheumatism; its Value and Mode of Action. By N. S. Davis, Jr., Chicago.

3. Acute Fatal Delirium; Its Differential Relations. By E. C. Spitzka, New York.

The nominations in this section are: for Chairman, Dr. H. B. Baker; for Secretary, Dr. Armstrong, of Memphis, Tenn.

---

### SECTION ON STATE MEDICINE.

In this section the following papers were read and discussed:

"The Necessity of Inspection of Food Animals." By Carl H. Horsch, New Hampshire.

"The Medical Climatology and Hydrology of Northern California." By J. W. Robertson, California.

"The Chemistry and Physiological Action of Tyrotoxicon." By Prof. V. C. Vaughn, University of Michigan.

---

### SECTION ON MEDICAL JURISPRUDENCE.

"Expert Testimony." By John Godfrey, U. S. M. H. S., Louisville, Ky.

"Criminal Responsibility of Epileptics." By H. M. Bannister, Kankakee, Ill.

"The Intimacy of the Medical and Clerical Professions, with the Supremacy of the Former in the Sociological Sense at Least." By Rev. L. T. Jones, Moweaqua, Ill.

"Forensic Relations of the Puerperal State." By H. C. B. Alexander, Chicago.

"Expert Testimony." By Wm. C. Wile, Philadelphia, Pa.

---

### SECTION ON OPHTHALMOLOGY, OTOLOGY AND LARYNGOLOGY.

In this section the following programme was carried out:

"Nature and Treatment of Phlyctenular Ophthalmia." By Dudley S. Reynolds, Louisville, Ky.

"Results of a Year's Experience in the Abandonment of the Eye Bandage." By J. J. Chisolm, Baltimore, Md.

"The Causitive Relations of Ametropia to

Ocular Disease." By J. E. Harper, Chicago.

"After-treatment of Cataract. Extraction." By W. T. Montgomery, Chicago.

"A Case of Epilepsy Apparently Cured by Correcting Hypermetropia," and "Two Cases of Tumor of the Optic Nerve." By Geo. E. Frothingham, Ann Arbor, Mich.

### THURSDAY'S PROCEEDINGS.

### THE GENERAL SESSION.

The Association was called to order at 10:10 a. m. Prayer was offered by Jno. H. Barrows, D. D.

The announcements of the Committee of Arrangements were made by Dr. Chas. Gilman Smith.

The Loyal Legion met at Kinsley's at 8 p. m., H. K. McKillop and B. K. Davis were made members by invitation.

The Ontario Medical Society sent a congratulatory telegram to the Association.

The report of the Nominating Committee was presented by Dr. Wm. Brodie, the chairman. The following officers were recommended to the association for election:

President—A. Y. P. Garnott, Washington, D. C.

First Vice-President—Duncan Eve, Nashville, Tenn.

Second Vice-President—Darwin Colville, New York.

Third Vice-President—Chas. J. O'Hagan, North Carolina.

Fourth Vice-President—A. Stedman, Colorado.

Librarian—C. H. Kleinschmidt, District of Columbia.

Treasurer—R. J. Dunglison, Pennsylvania.

Permanent Secretary—J. S. Ranschoff, Cincinnati.

Trustees of the Journal—L. Connor, Detroit; E. O. Shakespeare, Penn.; W. T. Briggs. Tenn.

Judicial Council—J. Murphy, Minnesota; J. M. Tonor, District of Columbia; J. R. Bartlett, Wisconsin; A. B. Sloane, Missouri; X. C. Scott, Ohio; A. W. McClure, Iowa; J. W. Stormant, Kansas. Vacancy occurring in the Council—J. F. Hubbard, Indiana.

COMMITTEE ON STATE MEDICINE.

Alabama—Jerome Cochran, Montgomery.
Arkansas—R. G. Jennings, Little Rock.
California—J. W. Robertson, Creston City.
Colorado—P. Brumund, Idaho Springs.
Connecticut—F. H. Whittmore, New Haven.
District of Columbia—G. W. Cook, Washington
Florida—U. D. Phillips, Gainesville.
Georgia—T. S. Hopkins, Thomasville.
Illinois—E. P. Cook, Mendota.
Indiana—T. B. Harvey, Indianapolis.
Iowa—Geo. F. Jenkins, Keokuk.
Kansas—W. L. Schenck, Osage City.
Kentucky—Dr. Larrabee, Louisville.
Louisiana—T. G. Richardson, New Orleans.
Maine—Dr. Foster, Portland.
Maryland—G. H. Rohe, Baltimore.
Massachusetts—Grace Walcott, Boston.
Michigan—A. Alvord, Battle Creek.
Minnesota—C. W. Hewett, Red Wing.
Mississippi—T. R. Trotter, Duck Hill.
Missouri—Lester Hall, Marshall.
Nebraska—Wm. M. Knapp, York.
North Carolina—Eugene Grissom, Raleigh.
New Hampshire—W. Porter, Walpole.
New Jersey—B. A. Watson, Jersey City.
New York—A. N. Bell, Brooklyn.
Ohio—F. C. Bain, Kentucky.
Pennsylvania—A. C. Dunn, Pittsburg.
Rhode Island—W. J. Berry, Pawtucket.
South Carolina—Thomas Ligare, Charleston.
Tennessee—Richard Cheatham, Nashville.
Texas—J. H. Sears, Waco.
Vermont—S. H. Griswold, Rutland.
Virginia—H. M. Nash, Norfolk.
West Virginia—P. J. Rowan, Grafton.
Wisconsin—J. L. Bartlett, Milwaukee.
United States Navy—Dr. Bloodgood.
United States Marine Hospital Service—C. B. Goldsborough.
Dakota—E. M. Darrow, Fargo.
New Mexico—Ross Bailey, Las Vegas.

COMMITTEE ON NECROLOGY.

District of Columbia—J. M. Toner, Chairman, Washington.
Maryland—John Morris, Baltimore.
Arkansas—T. E. Murrell, Little Rock.
North Carolina—J. H. Tucker, Henderson.
Vermont—M. R. Crain, Rutland.
Kentucky—J. D. Brooks, Paducah.
Dakota—J. N. Weir, Fargo.
Kansas—H. C. Minney, Topeka.
New Jersey—J. F. Ill, Newark.
Illinois—E. F. Ingalls, Chicago.
Tennesee—J. M. Savage, Nashville.
New York—L. D. Trowbridge, Palmyra.
South Carolina—F. L. Parker, Charleston.
Minnesota—A. W. Strickfield, Eyota.
Iowa—J. M. Emmert, Atlanta.
Alabama—B. L. Wyman, Tuscaloosa.
New Mexico—R. Bailey, Las Vegas.
Florida—K. A. Lancaster, Gainesville.
Indiana—J. H. Hibbard, Richmond.
Louisiana—R. Matas, New Orleans.
Missouri—Dr. Dalton, St. Louis.
Michigan—Geo. E. Ramsey, Lansing.
Pennsylvania—Frank Woodbury, Philadelphia.

Rhode Island—Wm. J. Burge, Pawtucket.
New Hampshire—J. W. Parsons, Portsmouth.
Massachusetts—S. J. Belt, Boston.
Virginia—L. Ashton, Falmouth.
Ohio—H. J. Herrick, Cleveland.
Wisconsin—J. T. Reeve, Appleton.
California—J. G. Terryl, Sacramento.
Texas—R. W. Park, Waco.
Colorado—M. H. Sears, Leadville.

The report of the Committee on Nominations was received and adopted. The Secretary announced the nominations of the following officers of sections.

Section of Diseases of Children—F. E. Waxham, Chicago, Chairman, and W. B. Lawrence of Batesville, Ark., Secretary.

Section on Oral and Dental Surgery—J. Taft, of Cincinnati, Chairman, and E. E. Talbot, of Chicago, Secretary.

Section on State Medicine—H. B. Baker, of Lansing, Mich., Chairman, S. T. Armstrong, of Memphis, Tenn., Secretary.

Section on Practice of Medicine, Materia Medica and Physiology—A. B. Palmer, Ann Arbor, Mich., Chairman, and N. S. Davis, Jr., Chicago, Secretary.

Section on Obstetrics and Diseases of Women—Eli Van De Warker, Syracuse, N. Y., Chairman, and E. W. Cushing, Boston, Secretary.

N. S. Davis gave notice that the Judiciary Council would meet at 9 o'clock at the Tremont House on Friday morning.

When the Section on Dermatology was called J. V. Shoemaker, of Philadelphia, offered his resignation. Various candidates were announced, but it was decided out of order and was referred to the section for new election. Dr. N. S. Davis said the adoption of the new amendments of the constitution made certain new machinery necessary, and among other things, the members from each state should meet and appoint two members to constitute a general committee, one to serve one year and one for two years, and this must be done to-day in order that the secretaries might report same to the association to-morrow. Dr. Bishop, of Pennsylvania, asked for information as to how it was that on yesterday the matter was spoken of as amendments to the by-laws, and to-day they appear as amendments to the constitution. He was in favor of the amendments, but did not favor the means used to carry them.

Dr. Davis replied that the report dealt, first of all, with amendments to the constitution, and after that with amendments to the by-laws, which had been distinctly stated in the report.

The President stating there being evidently a great deal of dissatisfaction concerning the report, and he desiring above all things to do right, he would decide that the amendments to the constitution would not be adopted, but laid over for a year, and the amendments to the by-laws would be adopted and go into effect at once.

Dr. Roberts, Tennessee, moved any further action in regard to the next meeting of the Association be deferred until May, 1888. Dr. Davis seconded the motion, and offered an amendment to the amendment to the by-laws, that the nominating committee give names of three persons to deliver addresses to the association next year in accordance with the new by-laws. After much confusion the matter was adopted.

Geo. H. Rohe, secretary of the Rush Monument committee, read the report, from which it appears that $389.00 had been collected, and $143.08 expended for printing and the like.

The report was accepted and referred to a committee consisting of Drs. Grissom, Garcelon and Palmer for auditing.

Dr. Ranney, of Michigan, stated at the last meeting of his State Society $100 was appropriated for this monument.

Dr. Keller, of Arkansas, chairman of the committee on cremation, was not present, and his report was read by Dr. Morris, of Baltimore. The report was as follows:

No facts of a practical character in addition to those so forcibly presented by the former committee have come to our notice during the year. A report made to the American Public Health Association at Toronto, last October, largely embodies the views of the committee. In that report it is stated that it is only in the case of sudden and violent symptoms of disease or a great epidemic that the failure of the ordinary modes of burial can be realized or properly brought to the notice of the people; that as long as such outbreaks do not occur no particular attention is given to the matter by the profession or the laity; that, inasmuch as cremation has not met with popular acceptance, a modified form of cremation called by Liebeg cremacaucis might be adopted. This looks to the adoption of municipal and state laws compelling the use of destructive agents to bring about the rapid disintegration of the dead body. Caustic, lime, or chloride of zinc are especially adapted to this office. This process of immediate destruction of the dead body is particularly desirable in cases of persons dying of zymotic diseases. The burial of persons dying of these diseases should be placed by law in the hands of the health authorities. The old-fashioned triple coffin and the vault should be entirely discarded. Earth-to-earth burial should, as far as possible, be encouraged. As our cities increase, as our populations thicken, the evils of our present mode of burial will increase. In the end it will be found that cremation is the truest, safest means of escape from the evils incident to the decomposition of the dead. The committee recommend the adoption of the following:

"Resolved, That it is the judgment of the American Medical Association that the burial of all persons dying of zymotic diseases should be placed by law under the control of the health authorities, and that in all such cases of disease chemical agents should be used by such authorities to bring about a rapid disintegration of the dead body."

Referred to the Committee on State Medicine.

Dr. Gaston introduced resolutions requesting the President to appoint additional physicians upon the yellow fever commission.

Dr. Rohe opposed the motion, but it was finally adopted.

The first paper read was the report of the Chairman of the Section on Obstetrics and Diseases of Women by F. M. Johnson, of Missouri.

Dr. Geo. H. Rohe, Chairman of the Section on State Medicine, read his paper on "Recent Advances in Preventive Medicine."

Dr. G. Knox, of Chicago, Chairman of the Section on Diseases of Children, read an address on "Diathesis and Disease in Early Life."

The report of the Treasurer was then read, showing the receipts during the past year had been $21,723.22, and expenditures $20,319.45, leaving a balance of $1,403.77.

The report was received and placed on file. It was moved and carried that the Society subscribe $10 for an Index Medicus.

The Librarian's report was received and adopted.

After considerable discussion the Association appropriated $1,000 for the International Medical Congress.

On motion the Society adjourned.

---

### SECTION ON DISEASES OF WOMEN AND CHILDREN.

Dr. H. Landis Getz, of Marshalltown, Iowa, read a paper on "Diphtheria." The author, stated that while this was a subject which had often been discussed by those having experience with the disease, many others had attempted to discuss it who had only had experience with "Ulcerated Tonsilitis," or what could be called "Homæopathic Diphtheria."

Admitting the disease to be of the zymotic class, highly infectious, slightly contagious, the chief early symptoms of which are malaise of a few hours' duration, with or without chills, followed by a slight fever. There is seldom vomiting or convulsions at this stage and the local symptoms are so slightly painful or inconvenient as to cause the inexperienced to overlook the real trouble.

Physicians are often called to determine the reason for not feeling well as late as twenty-four hours after the membrane formation has set in, which he believes is always found to exist as early as any noticeable fever. The severity of the attack cannot be usually discovered by the constitutional symptoms found to exist during the first thirty-six hours, but is determined alone by the soil in which it develops, the blood and other elements in some systems affording barren soil which renders them proof against constitutional contamination, while in others it is fertile, and becomes rapidly saturated unless active measures are taken to destroy the germs of disease.

Consequently two classes of remedies are required—germicides and fortifiers. The former may be applied locally and termed direct or indirect; the indirect so altering the character of the blood that the germs perish on reaching it.

Fortifiers are: 1st. Such remedies as place the tissues in such condition as to prevent the lodgment of the disease.

2d. The second class of germicides are used.

3d. Remedies, surroundings and elements which tend to strengthen the blood and system, rendering it a nearly barren soil for the disease.

As the disease is a rapid one, terminating usually in four or six days, the treatment must be prompt and thorough.

There can be little doubt concerning the seat of disease, and, if not constitutional at the outset, becomes so, and the treatment resolves itself into local and constitutional.

If treatment is commenced within twelve hours from the commencement of the formation there should be no fatal results; if twenty hours you must expect to lose a patient occasionally, but if twenty-four hours have elapsed you must expect many fatal results.

The malignity of the disease depends more on the quantity than the quality of the germs and in prevention of their development lies the secret of success in treatment.

Owing to the mildness of the early symptoms it becomes the duty of the physician to examine carefully when a patient has been exposed to diphtheria, or if there is any slight trouble in the throat whether patient has been exposed or not. In difficulties resulting from cold the local symptoms in the way of pain will call the attention to the organ.

There is much diversity of opinion as to whether membranous croup and croupal diphtheria are not the same disease, but, properly there is no such disease as diphtheritic croup (as croupal diphtheria is sometimes called), but it is diphtheria of a croup type involving an inflammatory condition and exudation of the larynx. The author gave a table showing that during a diphtheria epidemic in his city in 1886 there were 41 deaths (20 male and 21 female) out of something over one hundred cases reported, about one-third of the number being school children. The epidemic abated without any apparent changes in hygienic surroundings, water, etc., but the author thinks a factor for development of the disease exists in filth, and that, connected with certain atmospheric influences and tangible impurities, develops the disease.

The question of what remedies are best adapted in the way of drugs was answered—quinine and tinc. chlor. of iron. They are not new, but have not until recently been given the merit they deserve. In diphtheria the patient should, if an adult, have for first dose of quinine fifteen or twenty grains, subsequently 10 grains every 6 hours during the first 36 or 48 hours; after that enough to continue a slight ringing in the ears, care being taken to not overdose.

Tincture of iron may be used as a direct germicide and constitutionally as a fortifier by preserving the purity of the blood. It can be used as a local application varying in strength from one part of iron to three of glycerine and so on to full strength as the case requires. Applications should be made by physician personally, when possible, twice or three times in twenty-four hours. Twenty drops of the muriated tincture should be given adult in teaspoon of water every two hours as long as the stomach will stand it; if stomach becomes irritable lengthen the intervals. Hot applications are thought injurious, causing more rapid development of germs. Stimulants may be given when necessary. Ice may be used in the mouth. The main feature in the treatment should be even remembered by the physician, that to insure success, medicines, stimulants and food must be given on the hour and all local applications made by the physician himself.

Papers on the following subjects were read, in addition to the above:

" New Method of Treating Congenital Phymosis." By W. S. Stewart, Philadelphia, Pa.

" Intubation of the Larynx with Inferences from one hundred and thirty-three Cases." By F. E. Waxham.

### SECTION ON PRACTICE OF MEDICINE.

Dr. G. W. McCaskey of Ft. Wayne, Ind., read a paper on " A New Method of Intra-Pulmonary Medication" with remarks on its use for pneumatic treatment.

The author first elaborately reviewed the evidence tending to prove that medicinal agents can be introduced into the air cells with the inspired air, and then proceeded to discuss the question under what forms and conditions can various medical agents be introduced in such way as to insure their influence on the deep bronchial structures.

He was not in favor of powders, gases or sprays for introducing such medicinal agents. He relies on saturated vapors temperature 158°F., or in exceptional cases, a somewhat lower temperature. The air saturated at this temperature is about one-third vapor, and with a comparatively cool respiration the lung reduces the temperature to 113°F., and about 69 per cent. of its deposit would be vapor.

The author uses a special apparatus which was exhibited to the Section, and consists of a double walled chamber, capacity one cubic foot, the hot air of which can be saturated with the vapor of any soluble agent. Expiration is carried on continuously by a combination of valves; the patient inspiring from the chamber and expiring into the air of the room.

He uses a wet and dry bulb thermometer to determine when saturation is complete, and also to lower the maintenance of uniform temperature. Its use with the pneumatic cabinet the author believes to be of great advantage. He has had considerable experience in the treatment of bronchitis and phthisis and, apparently, has had more gratifying results than are usually obtained with the pneumatic cabinet alone.

The following papers were also read and discussed:

" A Convenient and Practical Method of Administering Burgeon's Treatment." By Byron H. Daggett, Buffalo, N. Y.

" New form of Apparatus for the Administration of Gaseous Enemata." By J. H. Kellogg, Battle Creek, Mich.

" Geranium Maculatum." By John V. Shoemaker, Philadelphia.

"Etiology of Typhoid Fever as Observed in Country Practice." By L. N. Davis, Farmland, Ind.

### BUSINESS IN THE OTHER SECTIONS.

The following programme of papers was carried out at the meetings of the various sections:

### SECTION ON OBSTETRICS AND DISEASES OF WOMEN.

"Intra-Uterine Therapeutics." By W. S. Caldwell, Freeport, Ill.

"Cystitis in the Female." By H. O. Marcy, Boston, Mass.

"Lithiasis in Pregnancy." By J. E. Kelley, New York, N. Y.

"Erosions vice Ulcerations of the Vagina. Portion and their Relation to Laceration of the Cervix, with Practical Hints when not to Perform Emmet's Operation." By John A. Miller, San Francisco, Cal.

"Sudden Death in Labor and Childbed." By Fayette Dunlap, Danville, Ky.

SECTION ON ANATOMY AND SURGERY.

"Laparotomy for Penetrating Wounds of the Abdomen." By T. A. McGraw, Detroit. (A lengthy abstract of this paper appears elsewhere.)

"Injuries of the Abdomen and their Proper Treatment." By N. B. Carson, St. Louis.

"Pathology, Diagnosis and Treatment of the Appendix Vermiformis." By J. McF. Gaston, Atlanta, Ga.

"Calculi in the Appendix Vermiformis." By R. H. Reed, Mansfield, Ohio.

"New Method of Testing the Relative Value of Certain Antiseptics, Disinfectants and Germicides employed in the treatment of Wounds and Syphilis." By Jos. Jones, New Orleans.

"Wound Dressing: Some Notions Accepted and Some under Discussion." By G. E. Stubbs, Philadelphia, Pa.

SECTION ON OPHTHALMOLOGY, OTOLOGY AND LARYNGOLOGY.

"Evulsion as a means for the Radical Cure of Pterygium." By J. W. Wright, Columbus, Ohio.

"Function of the Oblique Muscles in Certain Cases of Astigmatism." By G. C. Savage, Nashville, Tenn.

"Effects of Obliquity of the Correcting Lens to the Visual Axis." By Edward Jackson, Philadelphia, Pa.

"A New Form of Facial Frame." By Edward Jackson.

"Suppurative Inflammation of the Antrum of Heighmore." By E Fletcher Ingals, Chicago.

"Treatment of Hay Fever," and "Treatment of Chronic Suppurative Inflammation of the Middle Ear." By Seth S. Bishop, Chicago.

SECTION ON ORAL AND DENTAL SURGERY.

"Sponge-Grafting." By W. H. Atkinson.

SECTION ON STATE MEDICINE.

"Scientific Collective Investigation of Diseases." By H. B. Baker, Lansing, Mich.

"The Chemistry and Physiological Action of Tyrotoxicon." By V. C. Vaughn.

"An Inquiry as to the Mortality of Immigrants at United States Ports." By S. T. Armstrong, Memphis, Tenn.

"The Necessity for Inspection of Food Animals." By Dr. C. H. Horsch.

"Hygiene of Infancy and Childhood." By Dr. T. B. Greenley.

"Individual Hygiene of a Municipal Police Force." By Dr. G. Homan, St. Louis.

SECTION ON MEDICAL JURISPRUDENCE.

"The Medical Jurisprudence of Mental and Nervous Diseases." By S. V. Clevenger, Chicago.

"Paralytic Conditions in Relation to Testamentary Capacity." By E. C. Spitzka, New York.

"Report on the Present State of Our Knowledge concerning Concussion from Railway Accidents." By N. E. Brill, New York.

"A Claim to the Priority in the Determination of the Chemical and Microscopic Changes of the Blood in various forms of Malarial Paroxysmal Fever and the Application of the Results of these Investigations to Medical Diagnosis and Medical Jurisprudence." By Joseph Jones, New Orleans.

"Micrometric Measurement of Blood Corpuscles in Medico-Legal Cases." By Dr. Marshall D. Ewell, Chicago.

---

### FRIDAY MORNING'S SESSION.

The Association was called to order at 10:30 o'clock. President Gregory in the chair.

Prayer was offered by the Rev. W. H. Vibbert, D.D.

The report of the Committee of Arrangements was read by the Chairman, Dr. Smith.

The Nominating Committee, through Dr. Brodie, reported that R. Beverly Cole, of San Francisco, had been selected to deliver an address on "General Medicine" at the next meeting; E. N. Moore, of Rochester, N. Y., to deliver an address on "General Surgery," and J. L. Colell, of Virginia, to deliver an address on "Public Medicine."

The following committee was appointed to inform the gentlemen of their selection, and, if necessary, fill vacancies: J. N. Toner, of Washington; Eugene Grissom, of Raleigh, N. C., and Dr. Colvin, of Clyde, N. Y.

Dr. J. B. Hamilton introduced the following:

WHEREAS, The President of the United States has appointed George M. Sternberg, surgeon of the United States army, to proceed to Mexico and Brazil for the purpose of investigating the methods there practiced for the prevention of yellow fever by inoculation; and

WHEREAS, This report will be accompanied by photo-micrographic illustrations of the appearances of all the principal organs of the body affected by yellow fever; therefore, be it

*Resolved*, That the Senate and House of Representatives be requested to cause such number of copies of Dr. Sternberg's report to be printed as may be needed by the profession of medicine of the United States. Be it further

*Resolved*, That the resolution on this subject passed yesterday be rescinded.

Dr. Wilson, of Boston, seconded the resolution.

Dr. Gaston was opposed to the resolution, on the ground that the action taken on the resolution of the preceding day was final, the motion to reconsider having failed. He thought it impossible for the Association to rescind the action of yesterday.

Dr. Hamilton contended this was a new resolution. He did not propose rescinding any resolution; but, if in the body of the resolution presented there is anything that rescinds the action taken previously, it is clearly in order.

Upon request the resolution was read by the Secretary.

Dr. Rohe, of Baltimore, favored the passing of the resolution, saying that President Cleveland had already done all that could be asked of him. Dr. Sternberg had been appointed and was already at work. It would be ridiculous to allow the resolution of yesterday to stand as the sentiments of the Association. He hoped the Association would pass Dr. Hamilton's resolution in such a manner that there could be no doubt as to the opinion of the Association.

Dr. Gaston moved, and it was seconded, that the resolution be separated.

There was call for the previous question, and it was put, and the resolution of Dr. Hamilton carried by a large majority.

Dr. J. S. Marshall, Chairman of the Section on Oral and Dental Surgery, read his report, which was referred to the Committee on Publication.

Dr. J. N. Quimby, Chairman of the Section on Medical Jurisprudence, read his report, which was referred to the Committee on Publication.

Dr. J. N .Beel ,of New York, moved that committees referred to in Dr. Quimby's report be appointed.

The following gentlemen were named as a Committee on Criminality of Fœticide and Measures for its Prevention: J. N. Quimby, of New York; W. B. Atkinson, of Pennsylvania, and W. H. Byford, of Illinois.

Duties Commonly Exercised by Coroners: H. O. Marcy, Boston, Mass.; J. H. Burge, New York; W. W. Dawson, Ohio.

J. M. Toner, of Washington, read a brief report of the Committee on Necrology, which proposed to get out a necrological report for the last ten years.

The committee to audit the report of the Trustees of the Journal submitted their report, finding everything correct.

Dr. N. S. Davis presented the report of the Standing Committee on Meteorological Conditions and their Relations to the Prevalence of Disease; also concerning the Collective Investigation of Disease in Coöperation with the Committee of the British Medical Association. He reported progress, and, on motion, the committee was continued.

On motion, the report of the Chairman of the Section on Surgery and Anatomy was referred for publication.

Dr. N. S. Davis introduced the following resolution:

*Resolved*, That the regular graduates of such dental and oral schools and colleges as require of their students a standard of preliminary or general education and a term of professional study equal to the best class of the medical colleges of this country, and embrace in their curriculum all the fundamental branches of medicine, differing chiefly by substituting practical and clinical instruction in general medicine and surgery, be recognized as members of the regular profession of medicine, and eligible to membership in this Association on the same conditions and subject to the same regulations as other members.

In offering the resolution, he said he wished to explain the meaning of the resolution. The first object in the resolution is to relieve a degree of embarrassment that exists in the dental profession. Dental and oral surgery is as much a part of the field of medicine and surgery as ophthalmology or otology or any other ogology. Our mouths and teeth are as important as any other part of our organization, and are used a little more than any other part. The embarrassment arises in this respect: Just who and by what line those engaged in this department shall be recognized as members of the regular profession. Now it is proposed to make a line and draw it where this resolution says. There is a more far-reaching and more valuable underlying object. If this resolution passes this body it will raise the requirements of all the dental schools in the country so that they can be recognized as members of the regular profession of medicine.

The resolution was unanimously carried.

Dr. Davis then offered the following, which, after some debate, was adopted:

*Resolved*, That the committee of arrangements are hereby directed at each annual meeting of the Association to so arrange the programmes regarding entertainments and receptions that the evening of the third day be reserved for a regular annual dinner under the following general regulations: The chief registration officer shall provide for each registration table a paper headed,

"Annual Dinners of the American Medical Association," with two columns for names, one headed tickets without wines or liquors at a specified sum; the other, tickets with wines, etc., at a specified sum; that each member when registering can have the opportunity to take a ticket for the dinner if he desires it, and can be entirely free to enjoy the dinner not only without using wines, but, also, without being required to assist in paying for that drank by others; while those who desire the addition of wines will enjoy the same liberty. It shall be the duty of the committee of arrangements to select a proper place for the dinner, to ascertain the cost per plate on the plan already indicated, that the price paid for the tickets will pay the entire cost of the dinner, leaving no part to be paid either by the local profession or by the treasurer of the Association.

Dr. Wilson, of Boston, moved that an appropriation of $300 be given to the permanent Secretary. Dr. Davis objected to the motion, saying that the expenses of the Secretary were paid, and that every dollar that could be raised should be put into the Journal. The first year he had not a dollar, and since then it has been less than $1000 per year. He offered the following resolution as an amendment to the original resolution:

WHEREAS, It has been the unwavering policy of the Trustees for the publication of the Journal to enlarge and increase the value of the Journal as fast as the income of the Association will permit; therefore,

*Resolved*, That said Board of Trustees be a Standing Committee on Finance, to whom all propositions for the appropriation of money made hereafter shall be referred and reported upon before final action on the same by the Association.

Dr. Quimby—I rise to a point of order. There is a resolution before the house, and you cannot move a substitute.

Dr. Davis replied that his resolution was an amendment to the original resolution.

The motion was put on the amendment and carried. The original resolution failed.

Dr. J. N. Toner offered the following resolution as an amendment to the by-laws:

*Resolved*, That hereafter the Presidents of all such State and Territorial medical societies as are entitled to representation in the American Medical Association be recognized as Honorary Vice-Presidents of this body and entitled to seats upon the platform.

Dr. Jennings—It's a very fine thing in its appearance, but it would be rather over-doing the thing. While we honor the officers of the State associations, they are honored as much as they can be by becoming members of the American Medical Association. I can see no reason why the platform should be filled up with the officers of State societies. I think we have enough speakers on the platform now, for most of the business is transacted there.

Dr. Colvin—That might be very well for every State in the Union except New York. I should dislike very much to see the President of the New York State Medical Society on the platform.

Dr. Roberts moved to lay the resolution on the table. Carried.

Dr. Roberts moved that Dr. Brodie be made a committee of one to return thanks to those to whom thanks were due. Carried.

Dr. E. A. Wood introduced the following resolution:

*Resolved*, That a special committee of three be appointed to report on the subject of Dietetics at the next annual meeting.

Carried; and the following were named as the committee: Drs. Wood, Whittaker, and Woodbury.

The President appointed the following committee to draw up a form of law to secure uniformity in medical legislation, and to report to the Section on State Medicine next year: P. H. Millard, H. A. Johnson, R. H. Plummer, C. B. Belt, and C. W. Dullen.

Dr. T. E. Woodbridge introduced the following:

*Resolved*, That the President of this Association be requested to appoint a committee of three, whose duty it shall be to investigate thoroughly the whole subject of health resorts and mineral springs and report on their comparative value to this Association.

It was referred to the Section on State Medicine.

Dr. Brodie—It is always a good thing to be thankful after you have received something that you ought to be thankful for. I think we ought to be thankful for the beautiful Chicago weather we have had, and to the Committee of Arrangements, and especially to the Committee on Transportation for influencing the railroads to let us return on one-third fare. I think we should be especially thankful to THE CHICAGO MEDICAL JOURNAL AND EXAMINER for the full and complete reports we have had each morning. Of course we should be thankful to the Secretary, for he gets nothing but thanks. But then he is a rich man, any way—a pretty good fellow, whose expenses are all paid, and he makes out his own expense account. (Great laughter.) Finally, our especial thanks are due to our worthy President for the exceeding courtesy with which he has ruled over our deliberations. Gentlemen, we are thankful for everything.

President Gregory, in adjourning the meeting, said: "My work is finished—I wish it were done better. But it has been done as best I could. There is nothing remaining for me to do but to say, Thank you, good-bye, God bless you. I would gladly introduce to you your new President. But this is out of the question. I learn that he is a man—that he possesses those qualifications which will be in keeping with the men who have gone before him in this office. He is a man of culture, and endowed with those qualities of mind and heart that will insure success. I remember, thirteen months ago, the only man I envied was Dr. Brodie, the retiring President. I am proud to have been President, but I am glad that it is finished. It only remains for me, gentlemen, to add that you have honored me; you have trusted me; that you have been most deferential and indulgent, and if my heart had language it would gush. I must come to a close by offering my heartfelt thanks. The Society is adjourned to the second Tuesday in May, 1888."

## Figures and Tables

**Figure 1. f1:**
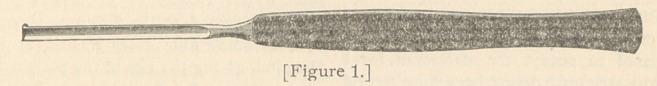


**Figure 2. f2:**
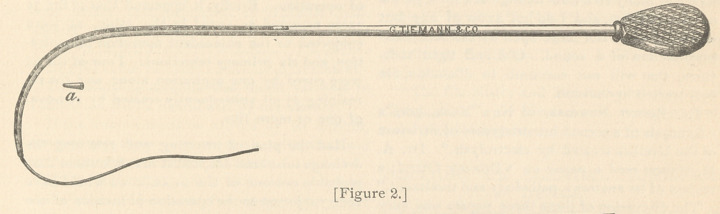


**Figure 3. f3:**
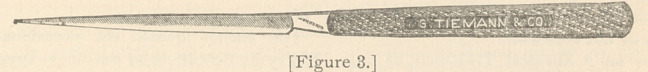


**Figure 4. f4:**
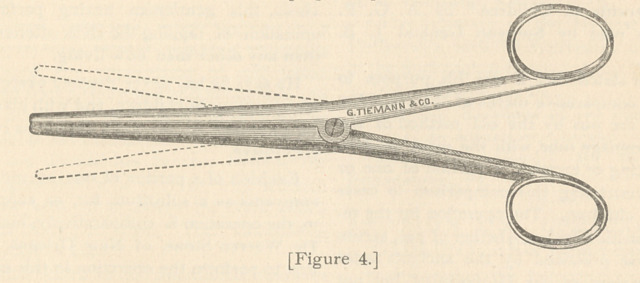


**Figure 5. f5:**